# Foxn1 in Skin Development, Homeostasis and Wound Healing

**DOI:** 10.3390/ijms19071956

**Published:** 2018-07-04

**Authors:** Joanna Bukowska, Marta Kopcewicz, Katarzyna Walendzik, Barbara Gawronska-Kozak

**Affiliations:** Institute of Animal Reproduction and Food Research, Polish Academy of Sciences, 10-748 Olsztyn, Poland; j.bukowska@pan.olsztyn.pl (J.B.); m.kopcewicz@pan.olsztyn.pl (M.K.); k.walendzik@pan.olsztyn.pl (K.W.)

**Keywords:** Foxn1, skin, development, wound healing, regeneration

## Abstract

Intensive research effort has focused on cellular and molecular mechanisms that regulate skin biology, including the phenomenon of scar-free skin healing during foetal life. Transcription factors are the key molecules that tune gene expression and either promote or suppress gene transcription. The epidermis is the source of transcription factors that regulate many functions of epidermal cells such as proliferation, differentiation, apoptosis, and migration. Furthermore, the activation of epidermal transcription factors also causes changes in the dermal compartment of the skin. This review focuses on the transcription factor Foxn1 and its role in skin biology. The regulatory function of Foxn1 in the skin relates to physiological (development and homeostasis) and pathological (skin wound healing) conditions. In particular, the pivotal role of Foxn1 in skin development and the acquisition of the adult skin phenotype, which coincides with losing the ability of scar-free healing, is discussed. Thus, genetic manipulations with Foxn1 expression, specifically those introducing conditional *Foxn1* silencing in a Foxn1+/+ organism or its knock-in in a Foxn1−/− model, may provide future perspectives for regenerative medicine.

## 1. Introduction

Skin, the largest organ in the body, acts as the outermost barrier to protect against water loss and a variety of external insults [1,2]. Furthermore, it has been shown that skin works as a complex, dynamic organ that is involved in neuro-immuno-endocrine function [1,2]. The cellular components of the epidermis are keratinocytes, melanocytes, Langerhans cells, and dendritic cells; those of the dermis are fibroblasts, dermal white adipocytes, vasculature and immune cell components. These components create an organ with tight communication among cells within and between the epidermis and dermis compartments [3].

Transcription factors are key molecules that tune gene expression and either promote or suppress gene transcription. The epidermis is the source of transcriptional factors, such as AP-1, Pparβ/δ, HoxA3, HoxD3, Smad2, Tcf/Lef (Wnt/β-catenin signalling pathway), Foxo1 and 3, Ovol1 and 2 [4,5,6,7,8,9], that are able to regulate many functions of epidermal cells, such as proliferation, differentiation, apoptosis, and migration. Furthermore, activation of epidermal transcription factors also causes changes in the dermal compartment, as was shown for Wnt/β-catenin signalling [8,10].

In this review, we focus on the transcription factor Foxn1, whose expression is restricted to organs with multilayered epithelia structures: the thymus [11,12] and skin [13,14,15,16]. The role and importance of Foxn1 in thymus development and function have been investigated intensively [11,17,18]. Although relatively less focus was accorded to the function and role of Foxn1 in the skin, growing evidence clearly indicates the significance of Foxn1 in skin development, homeostasis and wound healing [13,14,15,16,19,20,21].

The transcription factor *Foxn1* (forkhead box N1) gene, originally described as Whn (winged helix nude) or hfh11 (hepatocyte nuclear factor 3/forkhead homologue 11) was initially characterised in mice and rats [22] and later in humans [23].

Although the *Foxn1* gene was described relatively recently, mutations in this gene have been observed for a long time. In 1966, Flanagan published his observations of a new hairless strain of mice that had spontaneously arisen in the lab, which he called nude mice [24]. By crossing two normal-coated but presumably heterozygous mice, he established a stock of nude mice and showed that a recessive gene is responsible for the lack of fur.

Later, it was shown that an inactivating mutation in the *Foxn1* gene causes the nude phenotype, which is characterised by a lack of visible hair, abnormal development of the epidermis and a severe immunodeficiency due to thymus dysgenesis [22,25]. Immunodeficiency and the lack of fur make nude mice an excellent model for transplantation study, oncology research and skin permeability/penetration assays. Nude mice have received attention and serve as a model for skin wound healing to explain the role of T-lymphocytes in the healing process [26]. However, the study concerning the consequences of Foxn1 deficiency and the Foxn1 function/role in the skin occurred a decade later [13].

In this article, we review the current knowledge of Foxn1, focusing on its expression and role in the skin during development, homeostasis and wound repair.

## 2. Foxn1 in Skin Development

### 2.1. Foxn1 over Evolution Time

*Foxn1* is an evolutionarily ancient transcription factor maintained as a single copy in chordate genomes [22,27]. The *Foxn1* gene spans approximately 30 kb, and the locus is flanked by a sodium/dicarboxylate co-transporter gene positioned upstream and retinal gene 4 located downstream [28]. In both humans and mice, the *Foxn1* gene comprises eight coding exons; however, the first exon exists in two alternatives: exons 1a and 1b, each possessing a unique tissue-specific promoter. Accordingly, promoter 1a is active in the thymus and skin, whereas promoter 1b is present exclusively in the skin [28]. As other members of the forkhead/winged-helix class of transcription factors, Foxn1 is defined by a conserved, core DNA binding domain (DBD) that share at least 80% amino acid homology with distant metazoan representatives, including humans, bony fish, cartilaginous fish, agnathans and cephalochordates [29]. In contrast to amphioxi, in which the DBD is encoded by one large exon, in all investigated species, this motif is represented by three exons [29]. The DBD motif is approximately 80–100 amino acids in length and consists of three α-helices (H1–H3) connected via small β-strands (S1–S3) to a pair of “wings” (W1 and W2). Whereas helix 3 (H3) provides a canonical contact surface with the DNA target sequence 5′-TAAGTCA-3’, both wings are arranged along the adjacent DNA backbone and show higher variability patterns of interaction with DNA [30,31]. Although highly related across Foxn sub-families, DBDs have been demonstrated to be functionally different. The study by Schlake et al. [27] showed that replacement of the DBD in the mouse *Foxn1* gene with the human *FOXN2* sequence resulted in a lack of target gene activation. However, there is also evidence that the individual constructs carrying a mouse, zebrafish or amphioxus DBD expression plasmid activate transcription in baby hamster kidney and BHK cell lines to a similar extent. This finding suggests functional similarities among Foxn1 DBDs from the studied species [29]. Moreover, the structure of the Foxn1 protein is uniform across humans, mice and rats, revealing the presence of a transcriptional activation domain (AD) in the C-terminal region of Foxn1 that is essential for transcription onset as well as an N-terminal domain that is likely involved in protein-protein interactions [27].

Generally, transcription factors (TFs) and their regulatory networks have changed over evolutionary time. One of the underlying mechanisms has relied on gene duplications that result in expansion, diversification and divergence of existing TFs [32]. Interestingly, *Foxn1* genes were not subjected to genome-wide duplication; alternatively, these events may have occurred frequently and been followed by secondary losses [27,29]. Intriguingly, despite being exempted from gene multiplications, the expression of *Foxn1* has been constantly extended, and the gene has acquired a novel function. Several investigations have reported dynamics of the *Foxn1* expression site, indicating its primordial presence in gonads in the cephalochordate *B. lanceolatum*, its expression in fish eyes [27], and its detection in skin and hair follicles in mammals [13,16,19,33]. With regard to *Foxn1* specialisation and gain of a new function (in addition to its role in thymic epithelium differentiation), there is extensive literature demonstrating *Foxn1* involvement in normal keratinocyte and hair development, growth and differentiation [13,14,19,20,34,35,36], as well as in wound healing [16,21]. These topics will be discussed further in this review. In mammals, Foxn1 fulfils multiple functions consisting of engagement in unrelated differentiation programmes in the thymus and skin. It has been considered that the diversification of the role of Foxn1 arose from the changes in transcriptional control (*cis*-regulatory) sequences that occurred during evolution. Nevertheless, in lower vertebrates that possess a thymus but lack hair (chondrichthyes) or lack both structures (agnatha), the role of Foxn1 is questionable and leads to the conclusion that it may play different functions in chordates [29].

### 2.2. Foxn1 in the Development of Skin Tissue and Skin Appendages

#### 2.2.1. Foxn1 during Embryonic Development in Mice

A large body of data that refer to the pattern of *Foxn1* expression during murine development have originated from a single study presented by Lee et al. [19]. The authors employed transgenic mice in which an endogenous *Foxn1* allele was inactivated by the insertion of a *lacZ*-neomycin resistance cassette, resulting in the production of β-galactosidase that was previously shown by Nehls et al. to be a sufficient marker of *Foxn1* promoter activity [37]. The expression of the *Foxn1-lacZ* allele revealed spatial and temporal dynamics during heterozygous embryo development; it demonstrated its presence initially in the nasal region on the 13th day of gestation (E13) [19]. Next (E13.5), the expression was extended to the whisker pads and nail primordia, followed (E 14.5) by β-galactosidase activity that was detected in multiple regions, including the mouth, hair follicles (HFs) of the eyebrows and lashes, and the epidermis of the snout, ear and tail. Finally, at E15.5–16.5, *Foxn1-lacZ* allele expression spanned the entire epidermis and was induced in the developing hair follicles of the fur. With regard to the cell types expressing Foxn1, β-galactosidase activity was demonstrated in many types of epithelia, predominantly in suprabasal cells and occasionally in the basal layer. Within the epidermis, basal keratinocytes that are attached to the underlying basement membrane act as progenitors to suprabasal cells, which undergo terminal differentiation as they move towards the tissue surface. The first signs of this process have been reported from E14.5, which correlates with the induction of *keratin 1* (*Krt1*) expression in the epidermis at E15.5. Interestingly, this specific pattern of Foxn1 expression coincides with the transition from scar-free (regeneration) to scar-forming (repair) skin wound healing in a mouse and rat embryos that was established between day 16 and 17 of gestation [38,39,40]. Accordingly, genome-wide analysis of uninjured skin from a C57BL/6J (B6) embryo at day 14 of embryonic development (E14—model of skin regeneration) and E18 (model of skin repair) showed significant differences in gene expression between both models of healing, consisting of 2065 genes differentially regulated (up and down) [41]. Furthermore, the gene expression profile at E18 revealed much higher similarity to the profile of adult B6 mice, as only 96 genes were found to be differentially expressed. Importantly, in the same study, nude mice have been employed that, due to a loss-of-function mutation in *Foxn1*, exhibit a scar-less skin wound healing pattern that is recognised as regeneration [42,43]. Comparative analysis between both models of regeneration phenomena, adult nude mice and B6 foetuses, at E14 revealed a considerably lower number of differentially expressed genes (1451) than the number of genes that differed between adult nude (regenerative) and adult B6 (reparative) (2243 genes) [41]. Moreover, the analysis of genes similarly regulated in adult nude mice vs. B6 and B6 foetuses at E14 vs. B6 foetuses at E18 indicated that 193 up-regulated genes and as many as 465 down-regulated genes are common between nude and E14. A majority of down-regulated genes rather than up-regulated ones led to the conclusion that loss of gene activity facilitates the immature stage of skin development; thus, in part, it favours regeneration when injury occurs. The gene signature associated with lack of *Foxn1* in both the B6 embryo at E14 and the adult nude mouse is defined by an increase in genes involved in (1) tissue remodelling and extracellular matrix composition (*kallikreins*, *kallikrein-related peptidases*: *Klk7*, *Klk8*, *Klk10*, and *protease serine 27* (*Prss27)*; (2) *keratins* in the skin (*Krt23*, -*73*, -*82*, -*16*, -*17*) and epidermis (Krt6a and -*6b*); (3) cornified envelope formation, including *involucrin (Ivl), filaggrin (Flg2), small proline-rich protein 2d (Sprr2d)* and *repetin (Rptn)*; (4) the inflammation phase of wound healing (interleukin 1α (*Il-1A*), *Il-1 F6*, *Il-1 F8*, Il-18, *Il-5*, fibronectin 1 (*Fn1*); (5) scar healing (*tenascin* (*Tnc*); and decrease in (6) transcription factors associated with the homeodomain that act as transcriptional regulators and specifies cell fate during embryonic development and governs embryonic stem cell differentiation, such as *Hox* genes (*Hoxb3*, *Hoxb5*, *Hoxc6*, *Hoxd13*), *extended Hox* genes (*Gbx2*), *Paired domain* (*Pax1*), *Paired-like domain* (*Phox2b*, *Pitx1*), *Six/sine homeobox* (*Six1*, *Six2*) and *atypical Hox* genes (*Pknox1*); (full list of genes differentially expressed in nude and E14 is available in [41]).

#### 2.2.2. Foxn1 during Postnatal Development

The development of skin and its appendages, which begins during the prenatal period, continues to mature after birth. It has been long established that *Foxn1*, whose expression in skin is restricted to the interfollicular epidermis and hair shaft, is responsible for the initiation of terminal cell differentiation; thus, it encompasses the transition from proliferative to a post-mitotic stage [13,14,19].

In the maturing skin of mouse pups, *Foxn1* promotor activity has been reported in the first suprabasal layer of the epidermis and the supramatrical area of the hair bulb, which have been defined as regions associated with early stages of terminal differentiation [19]. However, Foxn1-positive cells have also been detected in the peripheral matrix and in single cells located in the basal epidermal compartment. Similarly, in a separate study performed on the intact skin of young (21–28 days), adult (2–3 months and 9 months), and old (16–18 months) Foxn1::Egfp transgenic mice, Foxn1 localisation has been demonstrated in suprabasal epithelial cells and HFs regardless of the animal’s age [21]. The similar levels of *Foxn1* mRNA expression in its host tissue, epidermis, was detected in young and adult mice, whereas the highest expression was observed in old mice. In contrast, flow cytometry analysis of keratinocytes isolated from the skin of Foxn1::Egfp mice showed comparable levels of the percentage of Foxn1-eGFP positive cells between adult and old mice and the highest percentage of Foxn1-eGFP positive cells in new-born littermates. Moreover, among Foxn1-eGFP-positive cells detected in the skin of new-born mice, the large population of cells exhibited markers typical of epithelial to mesenchymal transition (EMT) recognised as double positive E-cadherin/N-cadherin cells [16,21]. Considering developmental divergences between skin from Foxn1-positive (reparative) animals such as B6 mice and Foxn1-inactive/deficient (regenerative) nude mice [41], it has been necessary to analyse differentially regulated genes in the epidermis isolated from both animal models. Next-generation high-throughput DNA sequencing revealed that 306 genes were down-regulated, whereas 571 genes were up-regulated by 2-fold or more in the nude mouse epidermis. These evident number of differentially regulated genes that are potent targets of Foxn1 suggests that this transcription factor widely impacts the epidermis at the molecular and subsequently physiological levels [41].

Endogenous *Foxn1* expression in mouse interfollicular keratinocytes (epidermis) and follicular cells/compartment (HFs) is induced when epithelial cells lose the ability to multiply and enter terminal differentiation [13,14]. Indeed, *Foxn1* expression declines as differentiating keratinocytes travel from the first suprabasal layer towards the skin surface [19]. Additionally, in cultured mouse keratinocytes, Foxn1 levels dramatically increase following calcium treatment, which induces cell differentiation [20]. Multiple in vitro studies provide insight into the epidermal differentiation pattern and mechanism that drives the conversion of skin progenitor cells to differentiated phenotypes. Utilising mouse primary keratinocytes, Brissette et al. [13] showed that the expression of two late keratinocyte differentiation markers, involucrin and filaggrin, was substantially higher in cells derived from nude (Foxn1−/−) mice, while Krt1, an early differentiation marker, was present at lower levels in nude cells than in their wild-type counterparts. These findings, together with ex vivo data from Prowse et al. [14], indicate that Foxn1 regulates the transcription of genes related to the early stages of differentiation. Furthermore, Foxn1 simultaneously induces the proliferation of neighbouring cells to replace the differentiating population that exit the pool of progenitor cells [13]. Thus, Foxn1 maintains a balance between proliferation and differentiation in keratinocytes. Consistent with previous reports, Kur-Piotrowska et al. [44] demonstrated that the transient activation of Foxn1 in B6 mouse keratinocytes promotes the differentiation and activation of these cells, which are manifested by up-regulation of involucrin and Krt16, respectively.

The identification of pathways engaged in keratinocyte differentiation and simultaneously linked with Foxn1 activity and/or deficiency has been intensively investigated during the recent past.

Among leading candidates are the mitogen-activated protein kinase (MAPK) cascade [20], protein kinase C (PKC) [36,44], phosphoinositide 3-kinase (PI3-kinase) and their downstream effector serine-threonine kinase Akt [35,45,46]. As shown in an in vitro study, once cells commit to differentiation following calcium addition, the p42/p44 MAPK pathway is inactivated; this event correlates with Foxn1 induction, suggesting that these switches may be an early step towards the decision regarding whether to divide or differentiate. Notably, changes in MAPK activity controlling the balance between proliferation and differentiation are associated with their phosphorylation status instead of the total protein levels [20]. It has been considered that PKC is a key target of Foxn1 in keratinocyte fate determination; specifically, isoform PKCδ appears to be the most dependent on Foxn1 [36]. As reported by Li et al. [36], Foxn1 antagonises the effect of PKC in the skin. Thus, Foxn1 deficiency in nude mice correlates with excessive PKC signalling, resulting in cell differentiation abnormalities manifested by overproduction of late differentiation markers (loricrin, involucrin) and reduced expression of early markers (Krt10) [36]. However, supplementation with PKC inhibitors prevents late markers’ overexpression in primary cultures of nude keratinocytes, whereas the levels of early markers were corrected through the addition of more specific inhibitors targeting PKCα or PKCδ. Interestingly, the PKC involvement in the Foxn1 signalling pathway was also documented through pharmacological intervention. Nude (Foxn1 deficient) mice injected with immunosuppressant cyclosporine A (CsA) showed the restoration in hair growth. The study that followed revealed that this beneficial aspect of CsA treatment was due to its inhibitory effect on PKC activity. Since high levels of PKC expression in nude mice is caused by Foxn1 deficiency, CsA treatment substituted Foxn1 action in lowering PKC activity [25,47]. Microarray analysis performed on human keratinocytes transfected with a construct carrying human *FOXN1* cDNA revealed 32 upregulated genes, among which the Akt transcript was markedly induced [45]. Interestingly, in this model, FOXN1 increased Akt levels, although it initially suppressed its activity, which further led to Akt recovery. Moreover, transient transfection with FOXN1 (FOXN1ER) and Akt (myrAktER) retroviral constructs triggered the terminal differentiation of the reconstituted human epidermis. Nevertheless, the changes were dissimilar and indicated that the activation of FOXN1 induced early stages of differentiation but was insufficient to initiate the later sequences. In contrast, Akt activation correlated with late stages epidermal sheet differentiation [45]. According to recent data, retinoid-related orphan nuclear receptor α (RORα) serves as an upstream regulator of the Foxn1 gene since multiple consensus RORα response elements (ROREs) were found in FOXN1, and, in part, it functions as an integral element during differentiation in the epidermis [48]. In addition, *FOXN1* and *Notch1* mRNA expression was significantly upregulated by the increased expression of RORα4 and decreased by RORα silencing. The opposite approach that relied on the siRNA-mediated FOXN1 knockdown showed that FOXN1 downregulation prevented the ability of RORα4 to induce the expression of a number of differentiation marker genes in human primary keratinocytes. Combined, the data indicate a high level of complexity accompanying epithelial cell fate determination in the skin; in addition, they reflect the Foxn1 governing function within the greater system of regulators.

#### 2.2.3. Foxn1 in Hair Follicle Morphogenesis

HFs are contiguous with the surface epithelium and begin to develop as soon as specialised dermal cells condense and organise in clusters (dermal papilla); this, in turn, stimulates the overlying epithelial stem cells to grow downward and form HFs. Simultaneously, the inner layers differentiate into concentric cylinders to form the central hair shaft (HS) and the surrounding layer, which is recognised as the inner root sheath (IRS), while the outer root sheath (ORS) maintains contact with the basement membrane [49]. This event occurs between E15.5 and birth, whereas full maturation appears when bulb reaches the bottom of the dermis; it is established at approximately postnatal day 6 (P6) in mice [50]. Soon thereafter, highly proliferative matrix cells of the hair bulb produce progeny that terminally differentiate, elongate hair and push them to emerge from the skin surface. Finally, fully developed hairs comprise several concentric layers of cells with separate fates [49,51,52].

**Figure 1:**
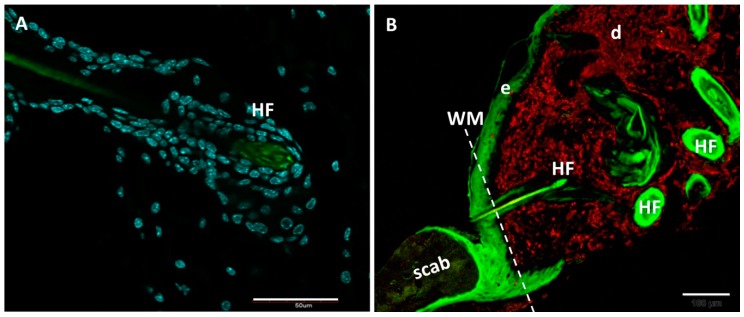
Histological analysis of skin sections collected from transgenic Foxn1::Egfp mice. (**A**) Confocal microscopy imaging of Foxn1/eGFP localisation in hair follicle (nuclei were counterstained with DAPI); (**B**) Fluorescent localisation of eGFP (green) and immunofluorescent detection of vimentin (red) in postwounded skin tissues. Foxn1-positive cells accumulated in the post-wounded skin area and in the leading epithelial tongue migrating underneath the scab. Abbreviations: WM—wound margin, HF—hair follicle, e—epidermis, d—dermis. Scale bar 50 µm (**A**), 100 µm (**B**).

Numerous data showed that Foxn1 is among the transcriptional regulatory proteins that participate in HFs progression during embryonic development and drives the hair cycle in neonatal and adult life [19,53,54] (Figure 1A).

In mouse embryos, the onset of *Foxn1* expression was reported to be induced in developing HFs of the fur coat on E15.5–16.5 [19]. Principally, follicular *Foxn1* was strongly expressed in a conical region above the bulbar matrix, which is the region that produces progenitor cells of the hair and IRS. Histological analysis of skin sections collected at days 1–2 after birth revealed the greatest *Foxn1* expression in the hair shaft. However, β-galactosidase, used as a marker of Foxn1 presence, showed its activity throughout the developing IRS and in a matrix compartment of the hair bulb, suggesting again that *Foxn1* correlates with the onset of terminal differentiation [19]. At postpartum day 9 (P9), Foxn1 protein was detected in differentiating precursors of the hair cortex that generate structural support for the HS and pigment from melanocytes [54]. As HFs complete their development, they undergo cyclic rounds of degeneration and regeneration throughout life. The pattern of *Foxn1* expression depends on the stage of the hair cycle. A study performed on one-month old *Foxn1* heterozygotes demonstrated the following pattern of *Foxn1* gene translation: (i) high levels in the first anagen (hair growth stage) were found in deeper parts of HFs; (ii) localisation restricted to the region neighbours with a developing keratinised structure of HS called “club” during catagen (regression stage) and (iii) low levels in the middle HFs segment (isthmus) in the telogen (resting phase) [19]. Similarly, mRNA microarray data from transgenic mice overexpressing miR-22 in the epidermal basal layer and ORS showed that, upon miR-22 induction, there were 74 genes downregulated during catagen and telogen. These genes included 27 keratin transcripts as well as their transcriptional regulators, including Foxn1, suggesting that the transcriptional programme regulated by Foxn1 may be controlled at post-transcriptional levels, specifically by microRNA [55].

Interaction between the epidermal layer and the underlying dermis is essential for skin homeostasis, HFs morphogenesis and cycling. Much attention has been paid to identifying pathways mediated by epidermal-mesenchymal communication. Although a large amount of data reveals that Wnt pathways play a vital role in this interaction [56,57,58], there is very limited literature documenting the action of Wnt signalling in concert with Foxn1 during HFs development and maintenance. As reported by Hu et al. Wnt5a, a direct Notch/CSL target in dermal papilla cells, regulates Foxn1 expression in HFs [59]. In the skin of mice with deletion of the Wnt5a gene or in reconstituted HFs with Wnt5a-null dermal papilla cells, Foxn1 expression was shown to be downregulated, while its levels increased in wild-type HFs upon Wnt5a administration. Moreover, transcriptional targets of Foxn1, including phospholipase C-δ1 (Plc-δ1) [60], desmocollin 2 (DSC2) [61] and trichohyalin, were down-modulated in Foxn1 knockdown HFs in vitro. Additionally, HFs isolated from transgenic mice exhibited deletion of the Notch nuclear mediator DNA-binding protein RBP-Jκ (CBF-1 or CSL). In contrast, treatment with exogenous Wnt5a induced expression of Foxn1-target genes in wild-type HFs with no activation observed in HFs derived from Foxn1−/− mice. Altogether, these data implicate the transcription factor Foxn1 as a mediator of RBP-Jk/Wnt5a activity in HFs.

#### 2.2.4. Foxn1 in Skin and Hair Pigmentation 

There is also strong evidence that Foxn1 participates in the development of skin epithelial cell coloration [54]. In regulating this process, in addition to external factors such as UV radiation, an important role is played by the communication between melanocytes and keratinocytes in the epidermis and fibroblasts in the dermis [62]. Specifically, in the pigmentation process, two different cell types are engaged: pigment donors (melanocytes) and pigment recipients (epithelial cells). Hence, in both humans and mice, Foxn1 marks pigment recipient populations. The synthesis of melanin begins within enzymatically directed melanosomes, which are present in the basal layer of epidermis. After reaching the state of maturity, i.e., fully melanised and low tyrosinase activity, melanosomes move from the perinuclear region to dendritic extensions. Using *Krt5-Foxn1* transgenic mice that lack Foxn1 in epithelial progenitor populations and nude (Foxn1−/−) littermates, Weiner et al. [54] proposed the mechanism underlying morphogenesis of pigmentary interactions between pigment donors and recipients in the hair cortex and other epithelia. Accordingly, Foxn1 activates epithelial cells to emit signals recognisable by melanocytes. One of the protein that contributes this signalling is fibroblast growth factor-2 (Fgf2), which exerts a potent effect on melanocytes in vitro [63]. Once melanocytes detect the mediators, they approve Foxn1-positive cells as targets, connect with them via dendrites and begin to transport pigment. Notably, the intracellular transportation of melanin to neighbouring keratinocytes occurs by formation of “the epidermal melanin unit” between melanocytes and keratinocytes. There are numerous mechanisms that are potentially involved in melanosomes transport. These mechanisms include exocytosis, cytophagocytosis, fusion of plasma membranes, and transfer via membrane vesicles, with the most likely being phagocytosis with the participation of protease-activated receptor-2 (PAR-2) and fibroblast growth factor receptor 2 (KGFR) expressed in keratinocytes [64,65,66]. However, in humans and mice, Foxn1 is commonly present in approximately 50% of the epithelial cells that receive pigment, suggesting the existence of Foxn1-independent pigment recipients.

#### 2.2.5. Foxn1 Deficient (Nude) Mice

One of the most informative examples providing evidence of how important Foxn1 is in skin epithelium homeostasis is Foxn1-deficient nude mice that exhibit multiple skin and hair defects. Nude mice, which carry a loss-of-function mutation in the *Foxn1* “nude” gene, were first described in 1966 by Flanagan [24] and have been extensively studied in the recent past. Mutations in the *Foxn1* gene were identified in the mouse allele *nu* and in two rat alleles, *rnu^r^* and *rnu* [22,67], located on chromosomes 11 and 10, in mice and rats, respectively (https://www.ncbi.nlm.nih.gov/gene). All of these genetic errors led to the formation of nonfunctional Foxn1 proteins. Notably, nude is a pleiotropic mutation since it is categorised into two independent phenotypic responses: (i) impaired skin keratinisation and aberrant HFs development [13,19] and (ii) thymus dysgenesis that leads to T-cell deficiency [37,68]. However, both of these abnormalities are not related to each other, since nude mice that received thymus grafts demonstrated the same mode of hair growth cycles and wave patterns as athymic nude individuals [69]. Moreover, lack of scarring (regeneration) when skin injury occurs, which is attributed to nude mice and associated with Foxn1 deficiency (see Paragraph 4.1), was not found in thymectomised neonates and their adult C57BL/6J counterparts or in immunodeficient mice: B6.129S7-Rag1tm1Mom and B6.CB17-Prkdc SCID/SzJ (SCID, severe combined immunodeficiency) [43], supporting the existing report that the lack of fur development and athymia have little in common. Considering that the Foxn1 protein is expressed exclusively in epithelial cells, exhibiting a critical role in interfollicular epidermal differentiation and control of HF lineages, nude mouse mutants represent multiple skin defects [13,19,70] (Table 1).

One possible explanation for the skin disorders associated with the nude phenotype is loss of the expression of the acidic hair keratin 3 gene (*mHA3*) in HFs, which acts under Foxn1 transcriptional control [33]. Indeed, the utilised nude skin at postpartum day 7 (P7) Schlake et al. conducted screening of the keratin mRNA expression [34]. Accordingly, not only *mHa3* but also *mHb5* is absent in Foxn1 null skin, indicating that both transcripts are direct Foxn1 targets.

Consistent with the ex vivo data, a study performed by Brissette et al. showed altered behaviour of nude keratinocytes in vitro [13]. Indeed, two late markers of keratinocyte differentiation, involucrin and filaggrin, were greatly elevated in Foxn1−/− (Swiss^nunu^) keratinocytes when compared with the wild-type. In contrast, Krt1, recognised as an early differentiation marker, was present at lower amounts in nude mouse primary keratinocytes cultured under basal and calcium-mediated differentiation conditions. Notably, Foxn1 was not only expressed in the skin (and thymus), but it was also observed in the epithelium of the nasal cavity, the palate, the tongue, teeth and nails [19]. However, malfunctions associated with nude mutation were found predominantly in nails, as these showed severe onychodystrophy and brachyonychia reflected by blunt, broken and irregularly ended nails; a thinned nail plate; an altered nail matrix; and loss of Krt1 expression and aberrant expression of filaggrin [71].

Phenotypic parallels between *Foxn1* inactive (nude) mice and *Hoxc13* gene-targeted transgenic animals (*Hoxc13^tm1Mrc^*) in accordance with DNA microarray analysis of skin from both models identified *Foxn1* as a significantly down-regulated molecule in addition to numerous genes of hair keratin and keratin-associated protein (KAP) [74]. Accordingly, the authors propose that the Hoxc13, Foxn1 and keratin/KAP connection works as a regulatory network motif known in the literature as a coherent feed-forward loop (FFL) [75]. This regulatory circuit found in biological systems relies on transcription factor X, which regulates transcription factor Y, and both regulate gene Z in a characteristic manner such that the signs of paths of gene Z regulation are the same [75]. In this context, transcription factor LIM homeobox 2 (Lhx2), an epithelial cell marker necessary for hair cycle progression, might be considered a potential candidate that contributes a loop with Foxn1 during HF development. Indeed, as reported by Bohr et al. [76], Lhx2 was constitutively induced in nude mice within the bulge region and interfollicular epithelium, as opposed to control animals. In wild-type mice, the induction of a new hair cycle and entering early anagen following depilation was associated with increased expression of embryonic stem cell markers, Sox2 and Oct4, while in nude mice, these markers in concert with *Krt14* and *Krt15* transcripts were downregulated, suggesting that upregulation of Lhx2 led to blockage of the early stage of progenitor differentiation: hence, Lhx2 acts upstream of Foxn1 transcriptional control. These findings also provide evidence for Foxn1’s possible role in controlling the epithelial stem cell fate. A study by Cai et al. demonstrated the genetic relationship between *Foxn1* and another transcription factor, homeobox protein Msx2, which acts upstream of *Foxn1,* since *Foxn1* expression is reduced in *Msx2* mutant HFs [77]. The Msx2 controls the expression of cortical keratins and KAP; hence, its mutant demonstrated downregulation of keratin transcripts. Although *Foxn1* mutant mice demonstrate more severe hair defects than their *Msx2* counterparts, the most severe differentiation defects of HS and IRS were found in *Msx2*/*Foxn1* double mutant HFs. Additionally, as double mutants showed a phenotype similar to that observed in *BMPR1a-*deficient mice, this leads to the suggestion that both Msx2 and Foxn1 act downstream of BMP signalling in HFs [77].

#### 2.2.6. Foxn1 Deficient Human Nude/SCID Phenotype

The human equivalent of the mouse nude phenotype was reported for the first time in two sisters in southern Italy in 1996 [23]. The affected subjects demonstrated complete alopecia of the scalp, eyebrow, and eyelashes and nail dystrophy associated with a primary severe T-cell immunodeficiency. However, the presence of phenotypically mature T cells (CD3^+^) and a lack of their immature (CD1^+^) counterparts, together with helper T lymphocyte (CD4^+^) deficiency, indicated the qualitative nature of the disease caused predominantly by deprivation of T-cell function. An in utero study performed on human FOXN1-deficient foetuses showed that alteration preventing T-cell development appeared as early as 11 postmenstrual weeks of gestation [78]. Further genetic evaluation of the human *FOXN1* gene revealed a homozygous C-to-T transition (CGA to TGA) at nucleotide position 792, which results in a nonsense mutation at residue 255 (R255X) in exon 5 [70]. Consequently, the translated FOXN1 protein is non-functional. Although skin of humans and rodents represents multiply differences in its structure [79,80] and the pattern of homeostasis restoration [81], FOXN1 deficiency in humans leads to defects similar to that observed in mice [24] and rats [22] and is recognised as a novel form of SCID named the Nude/SCID phenotype [82]. Since the first publication by Pignata et al. [23], only a few investigations of human nude phenotype have been published to date [72,73].

#### 2.2.7. Foxn1 Transgenic Animal Models

Spontaneous loss-of-function mutation in *Foxn1* gene results in a well-described nude phenotype associated with abnormalities in cutaneous and thymic epithelial development. In recent years, there has been great progress in identifying consequences of ectopic *Foxn1* expression. Prowse et al. introduced transgenic *inv-whn* mice expressed Foxn1 under the involucrin (late stage epidermal differentiation marker) promoter [14]. The animals were divided into groups depending on the exhibited phenotype. Accordingly, 72% demonstrated the “severe phenotype”, which caused death shortly after birth, whereas the second group displayed “mild effects” and survived to adulthood. Representatives of the severely affected group suffered from skin dehydration, which manifested as flaky skin and dramatic weight loss over several hours of birth. Histological analysis revealed multiple skin defects in transgenic mice, including hyperplasia of basal epidermal layer, lack of clear dermal/epidermal border, lack of progressive flattening in the suprabasal layers and increased keratinocytes vacuolisation. Adult mice of the mildly affected group showed aberrant hair production, which was reflected by sparse, bent hair with areas of alopecia. Moreover, hair displayed an inability to undergo a hair cycle; specifically, HFs were arrested in anagen and produced hair continuously. Finally, although transgenic animals survived to adulthood, adult individuals showed increased morbidity and mortality. Thus, it is worth noting that transgene expression lies in two major effects: (i) epithelial cell hyperplasia resulted in epidermal thickening and extended hair growth; (ii) disruption of keratinocyte and HFs terminal differentiation manifested by flaky skin and anagen-arrest HFs. However, suprabasal keratinocytes expressed Krt1 and Krt5, indicating that initiation of terminal differentiation occurs but the cells maintain basal-like features improperly. Altogether, these data indicate that Foxn1 is an important molecular player acting as a regulator of skin epithelia homeostasis, as gain-of-function (*inv-whn* transgene) and loss-of-function (nude phenotype) mutations both result in vast defects of keratinocyte growth and differentiation [14]. Similarly, *FoxN1^Tg^K14Cre^+^* transgenic mice with inducible *Foxn1* expression shared a similar phenotype with *inv-whn* transgenic rodents recognised by open eyes at birth and neonatal lethality caused by dehydration. Indeed, Foxn1 overexpression in Krt14+ epithelial cells led to increased permeability in parallel with the enlargement of epidermal thickness in transgenic new-born skin [83]. Again, consistent with the previous report [14], these data revealed that Foxn1 over- and ectopic expression might lead to a pathological effect in the skin. These findings also led to the conclusion that sustainable skin development requires the appropriate Foxn1 levels between two extremes: its deficiency and overexpression.

## 3. Foxn1 in Mature Skin Homeostasis

### 3.1. Foxn1 Localisation and Expression

The first report regarding Foxn1 localisation in the skin was made by Lee et al. [19]. Histochemical analysis of tissue isolated from transgenic mice, in which a β-galactosidase reporter gene was placed under the control of the wild-type *Foxn1* promoter, revealed that, in the skin of Foxn1+/− and nude Foxn1−/− mice, β-galactosidase-positive cells appeared in the suprabasal layer but also sporadically in the cells of the basal layer [19]. A recent study employing Foxn1::Egfp transgenic mice, in which the enhanced green fluorescent protein (eGFP) transgene is driven by *Foxn1* regulatory sequence confirmed Foxn1 positivity in the interfollicular epidermis and HFs [16,21]. Epidermal localisation of Foxn1 was detected in keratinocytes in suprabasal layers and occasionally in keratinocytes in basal layers. Immunofluorescent skin analysis showed co-expression of Foxn1 with E-cadherin (marker of epithelium) in all layers except the basal layer, in which the eGFP signal was observed sporadically [16]. Immunophenotypic analysis of Foxn1-eGFP cells revealed that the majority (60%) are E-cadherin positive. Moreover, within Foxn1-eGFP population, a small subset of cells (3.5%) displayed markers of both epidermal and dermal origin (E-cadherin/N-cadherin positivity) [16].

### 3.2. The Role of Foxn1 in Epidermis: Proliferation, Differentiation and Apoptosis

As noted, keratinocytes in the basal layer of epidermis have proliferative properties; however, when separated from the basement membrane, they enter a strictly controlled terminal differentiation programme [13,14,19]. A study performed on wild type and transgenic mice in which an endogenous *whn* (*Foxn1*) allele was inactivated by the insertion of a β-galactosidase *lacZ*neomycin resistance cassette showed that Foxn1 expression is induced in suprabasal layer of epidermis (epithelial cells that enter the differentiation programme) and occasionally in the basal layer [19]. To further investigate Foxn1 function, Prowse et al., generated transgenic mice that express *whn* under the involucrin promoter [14]. The distribution of Ki-67-positive keratinocytes (marking proliferative stage) were more frequent in transgenic (*inv-whn*) than wild type mice. Ki-67 positive keratinocytes localised primarily to the basal layer of epidermis. It was concluded that ectopic Foxn1 expression in differentiating cells (suprabasal layer) induces proliferation of neighbouring (basal layer) epithelial cells through a paracrine mechanism [14]. Recent in vitro data from Kur-Piotrowska et al. demonstrated that Ad-Foxn1 transfection in the primary keratinocytes decreased the percentage of keratin 14 (markers of basal (mitotically active)-positive cells and an increase in keratin 10 (markers of suprabasal, differentiated cells) positive cells which corresponds to the reduction of mitotic ability of Foxn1-overexpressing cells [44].

There is also evidence that Foxn1 in keratinocytes is involved in the regulation of apoptosis; however, the results of the studies are ambiguous [45,46]. Human keratinocytes transduced with FOXN1ER and stained with terminal deoxynucleotidyl transferase dUTP nick end labelling (TUNEL) did not revealed changes in the number of TUNEL-positive cells, showing a lack of correlation between the presence of Foxn1 and activation of apoptosis [45]. Further studies by this group suggested that Akt regulates caspase activity in the initiation and progression of differentiation process of keratinocytes, which is an altered form of apoptosis [46]. Considering that terminal differentiation has similar characteristics to apoptosis and that Foxn1 has an inhibitory effect on Akt levels, this may suggest the involvement of Foxn1 in the process of apoptosis [46]. The study by Kur-Piotrowska et al. employing Foxn1 overexpression in primary culture of mice keratinocytes, followed by proteomics analysis revealed changes in proteins with pro-apoptotic and anti-apoptotic features [44]. Interestingly, Foxn1-overexpression up-regulated levels of pro-apoptotic proteins (cathepsin D precursor (Ctsd), prolyl 4-hydroxylase (P4hb), galectin 7 (Lgals7) and voltage-dependent anion-selective channel protein 1 (Vdac1) and concomitantly stimulated production of pro-survival proteins such as heat shock protein 70 (Hsp70) and thioredoxin (Txn). Ensuing experiments employing flow cytometry analysis of cell cycle and apoptosis showed a decrease in the viability of cells and a higher number of apoptotic cells in Ad-Foxn1 keratinocytes. Furthermore, cells overexpressing Foxn1 accumulated in a G0/G1 phase, which may suggest the domination of non-proliferative cells in this population [44]. The data imply that the transcription factor Foxn1 may play multiple roles in cell fate determination/programming by regulation processes of proliferation, differentiation and apoptosis.

### 3.3. Age Related Foxn1 Modulation

There are limited studies related to the modulation of Foxn1 expression in the skin with possible correlation to animal age. A recent report by Kopcewicz et al. showed the highest *Foxn1* mRNA expression in enzymatically separated epidermis (the host tissue of Foxn1 expression in the skin) collected from old (16–18 months) animals when compared with young (21–28 days) and adult (2–3 and 9 months old) C57BL/6 mice. These data are in contrast to the thymic tissues, where the levels of *Foxn1* mRNA expression declined with animal age [21]. However, comparative flow cytometry analysis of the percentage of Foxn1-eGFP positive skin cells among new-born, adult and old B6 mice revealed the highest number of Foxn1-eGFP^+^cells in the group of new-born mice and a significantly lower percentage in adult and old individuals [21]. Phenotypic characterisation of Foxn1-eGFP-positive cells showed that approximately 55%, regardless of animal age co-expressed the epithelial marker E-cadherin. However, skin isolates from new-born mice displayed greatly elevated percentages of E-cadherin/N-cadherin (epithelial-to-mesenchymal marker) cells (23.75%; *p* < 0.01) within the Foxn1-eGFP cell population. The result was four- and twofold higher than those of the groups of adult and old animals, respectively [21]. The presence of E-cadherin/N-cadherin double positive cells may indicate the EMT process, as suggested by Kong et al. in studies of murine skin between embryonic day 18.5 and postnatal day 9 [84]. Moreover, the E-cadherin/N-cadherin and Foxn1 co-expression implies the possible involvement of Foxn1 in EMT.

### 3.4. Foxn1 Regulation

Comparative analysis of the gene transcript expression profiles of the skin of nude (Foxn1 negative) and control (Foxn1 positive) mice suggest an interaction between the Foxn1 transcription factor and genes associated with low oxygen availability [41]. In the skin of nude mice, low levels of gene expression related to hypoxia (*Hif-1α*, *Hif-2β*, *Hif-1 inhibitory factor (FIH1*) were detected. In particular, downregulation of Hif-1α and Hif-2β, which have a significant role in the control of transcriptional activity in the epidermis [85], points to the connection between Foxn1 and hypoxia-related genes. In addition, proteomics analysis of keratinocytes shows, among proteins that are up-regulated by overexpression of Foxn1, a distinguished group of proteins involved in Hif1α-/hypoxia-activated pathways (Txn, Hsp70) [44]. Furthermore, Foxn1 transfection into keratinocytes induced Txn, the main free radical scavengers, which enhances Hif1α protein levels and increases the expression of P4hb responsible for Hif1α degradation and Ctsd involved in Txn degradation [44]. However, Foxn1-overexpression also stimulated proteins related to/present in normoxic conditions (P4hb, Ctsd, mitochondrial aconitate (Aco2), enoyl-CoA hydratase, mitochondrial precursor (Echs1). The data indicated the role of the transcription factor Foxn1 in the cell response to hypoxic condition. Studies performed on keratinocytes isolated from B6 mice (endogenous Foxn1 expression) and from nude mice transfected with Foxn1 adenovirus (exogenous Foxn1 expression) cultured under hypoxic (1% O_2_) or normoxic (21% O_2_) conditions showed highly significant increases in Foxn1 expression in both types of cells exclusively under a hypoxic environment. Moreover, in the same study, it was confirmed that Mmp-9 is upregulated by hypoxic conditions. Collectively, the data strongly suggest that, stimulated by low oxygen availability, Foxn1 protects and allows cells to adapt to hypoxic conditions [44].

### 3.5. Foxn1 and Dermal Compartment of the Skin

Precise communication between the epidermis and dermis is essential for skin development, skin morphogenesis, and maintenance of skin homeostasis. Keratinocytes and fibroblasts-delivered factors are responsible for establishment of the basement membrane [86]. Moreover, both types of cells based on a double paracrine mechanism control the growth and differentiation of keratinocytes [87]. At the same time, epidermal signalling modulates the fibroblasts phenotype in the dermal layer of the skin, as was shown for the Wnt/β-catenin pathway [10].

The possible role of the epidermal transcription factor Foxn1 in dermal fibroblasts phenotype determination was analysed through comparison of fibroblasts (DFs) from nude (Foxn1 deficient) and wild-type (Foxn1 active) mice [47]. Flow cytometry analysis showed a higher percentage of cells expressing stem cell markers, including CD117 and Oct3/4, collected from the skin of nude mice (Foxn1 deficient) than from that of B6 mice (Foxn1 active) [47]. Additional changes were also observed in the expression of collagen I and III. In nude dermal fibroblasts, mRNA levels of *collagen I* and *III* were higher than those in DFs isolated from B6 mice. Increased production and higher levels of matrix metalloproteinases 3, 9 and 13 were also observed in DFs isolated from nude mice [47]. Moreover, fibroblast growth factor-2 (Fgf-2) in cultured cells induced expression of Mmp-3, -9 and -13 exclusively in nude DFs [41].

Fibroblasts from nude mice also showed increased plasticity in comparison to their wild-type counterparts, as manifested by greater differentiation capacity into adipocytes and an increase in cumulative population doubling when seeded at a low plating density [88]. Thus, the cellular features of nude fibroblast may be similar to those observed in mesenchymal stem cells, indicating their neonatal, regenerative capacity. Furthermore, the data showed that DFs phenotype is affected by factors secreted by keratinocytes [88]. The conditioned medium from Foxn1-active keratinocytes increased the metabolic activity of DFs from wild-type mice, while the medium from nude (Foxn1-nonactive) keratinocytes exerted the opposite effect, which is manifested by the reduction in DFs viability [88]. Therefore, the obtained results indicate that Foxn1 expression, although limited to the epidermis, may also impact the DFs phenotype.

Interestingly, DFs from nude mice revealed higher sensitivity to cyclosporine A, indicating the differences in DFs between nude and B6 mice, as well as the different effects of CsA on these two fibroblast populations. Treatment with CsA reduces the expression of Mmps and collagen III in dermal fibroblasts from regenerative (nude) mice to the levels observed in non-regenerative (wild-type) animals. Moreover, CsA treatment retarded the migratory ability of DFs, particularly those isolated from nude (regenerative) mice. These data suggest that CsA treatment in the same degree substitutes for Foxn1 activity, implying their inhibitory effect on the regeneration process [47].

An interesting issue is the possible contribution of the transcription factor Foxn1 in the lipid metabolism in the skin. Studies by Lanzini et al. using the liquid chromatography/high-resolution mass spectrometry (LC/HRMS) method allowed determination of lipid changes in the skin of nude versus BALB/c mice [89]. In Foxn1-deficient (nude) mice, increased levels of cholesterol sulphate, phospholipids, sphingolipids and fatty acids were detected [89]. The lipids that were found in the outermost layer of the epidermis are the components of the epidermal barrier that protects against external factors, prevents excessive water loss and participates in the regulation of thermogenesis. Moreover, analysis of the next-generation sequencing data obtained from nude vs. B6 skin and epidermis by Kur-Piotrowska et al. showed differences in the expression of genes involved in the development and modulation of adipose tissue, the synthesis of fatty acids and the absorption of cholesterol, indicating a possible role of Foxn1 in these processes [41]. Furthermore, the *in vivo* experiments housing nude mice under mild cold stress conditions (23 °C) showed the inability of these mice to gain weight due to diet-induced obesity [90]. Interestingly, the same effect of not gaining weight due to an obesogenic diet was observed in mice with inactive phospholipase Cδ1 (PLC-δ1) [91]. PLC-δ1 KO mice share similar to nude mice skin phenotype features. In vitro studies on U2OS osteosarcoma cells transfected with Foxn1 demonstrate increased PLC-δ1 at mRNA and protein levels, strongly suggesting regulation of this gene by Foxn1 in the skin [60]. Comparative analysis of gene expression between skin transcripts of nude mice and control mice with proteomic analysis of keratinocytes overexpressing Foxn1 further indicates that phospholipase Cδ1 is a target of Foxn1 [41,44].

Increased attention has recently been paid to dermal white adipose tissue (dWAT) [92,93,94,95]. Data from Donati et al. [8] and Mastrogiannaki et al. [96] indicate that epidermal Wnt/β-catenin is a critical initiator of the signalling cascade that induces adipogenesis in the skin, and its deregulation potentially contributes to the pathogenesis of fibrotic skin diseases. Considering the common expression of Wnt and Foxn1 in the epidermis, the involvement of both in the regulation of dermal component activity and the reports indicating that secreted Wnt glycoproteins regulate Foxn1 expression [68] it seems reasonable to hypothesise the role of Foxn1 in dWAT physiology. Whether Foxn1 activity/non-activity in the skin affects dWAT is currently under investigation.

## 4. Foxn1 in Skin Wound Healing

Cutaneous healing is a highly orchestrated physiological process that leads to the reconstitution of the integrity of the skin, which is a protective barrier against threats from the environment [97]. The response to the injury can be viewed according to the final outcome of this process: scar-forming wound repair or scar-free regeneration.

In mammals, the scar-forming wound healing process occurs in three overlapping phases: inflammation, new tissue formation and remodelling [98]. During the first stage of wound healing, inflammatory pathways and the immune system are needed to remove debris and prevent infection. The second stage of wound repair, the new tissue formation, is characterised by proliferation and migration of different cell types. Keratinocytes proliferate and migrate over the injured dermis to re-establish the skin barrier (re-epithelialisation), whereas dermal fibroblasts refill the wound gap. The extracellular matrix (ECM) is synthesised by interacting fibroblasts and myofibroblasts. During the last stage of wound repair, remodelling, the acellular matrix, composed mainly of type III collagen, is reorganised into scar tissue. Secreted by dermal fibroblasts, matrix metalloproteinases are involved in remodelling by proteolytic degradation of ECM proteins. A mature scar consists predominantly of type I collagen and never attains the same breaking strength and functionality as uninjured skin [98,99,100].

### 4.1. Scar-Less Skin Wound Healing in Foxn1 Deficient (Nude) Mice

**Scheme 1:**
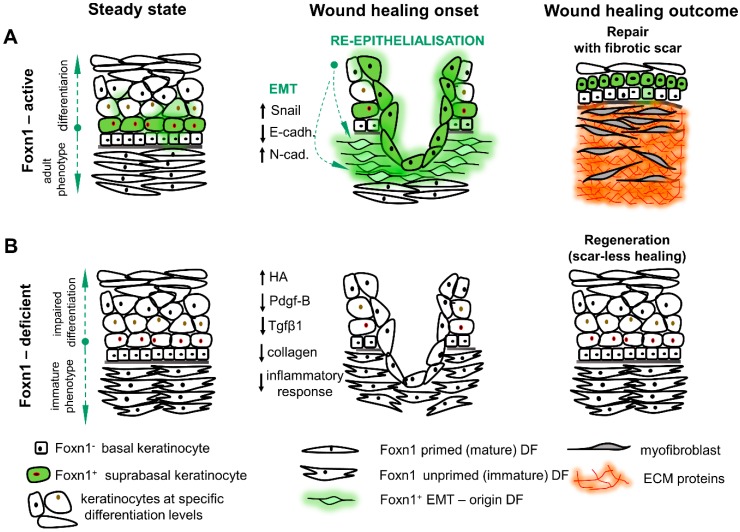
The scheme illustrates Foxn1 skin localisation and its possible role at steady state and during cutaneous wound healing. (**A**) The Foxn1 expression in the skin of Foxn1+/+ mice is limited to the suprabasal keratinocytes (green colour), and its action comprises the early stages of keratinocyte differentiation as they move towards the skin surface. However, the Foxn1 action extends beyond the host tissue (epidermis) to the underlying dermal layer. During wound healing, Foxn1 is principally engaged in re-epithelialisation and EMT processes, as manifested by its increase in Mmp-9 activity, Snail and E-cad/N-cad double-positive cell expression (green colour). The process leads to reparative healing occurring with fibrosis and scar formation, a condition characterised by excessive deposition of ECM (red colour); (**B**) Loss-of-function mutation in the Foxn1 gene results in impaired keratinocytes differentiation and immature (neotenic) skin phenotype; thus, it provides conditions favourable to regeneration when injury occurs. Scar-less healing is accompanied by high levels of hyaluronic acid, low levels of collagen and pro-scarring cytokines (Pdgf-B and Tgfβ1), and a unique, bimodal pattern of Mmp-9 and Mmp-13 expression. Abbreviations: EMT—epithelial to mesenchymal transition; Mmp—matrix metalloproteinase; E-cad—E cadherin; N-cad—N cadherin; ECM extracellular matrix; HA—hyaluronic acid; Pdgf-B—platelet derived growth factor B; Tgfβ1—transforming growth factor β1; DF—dermal fibroblast.

The general consensus is that adult mammals are not capable of regeneration. However, even in mammals, there are some well-described exceptions. Through-and-through punches made in the external ears of rabbits and Murphy Roths Large (MRL/Mp) and the closely related Murphy Roths Large/lymphoproliferative (lpr) (MRL/MpJ-Faslpr) mice heal in a process resembling regeneration [101,102,103]. Studies by Gawronska-Kozak et al. revealed another example of adult mammals with enhanced ability for scar-free healing, namely, Foxn1-deficient (nude) mice [43,104]. Similar to rabbits and MRL mice, Foxn1-deficient mice re-grow holes punched into their ears in a regenerative manner. The rapid re-epithelialisation process after wounding is followed by the formation of blastemal structure, and dermal, vascular, cartilage and muscular regenerative outgrowth [104]. Further studies showed that nude mice are able to heal skin incisions without or with nearly invisible scar formation [43]. The experiments performed on C57Bl/6J (wild-type controls), nude (B6.Cg—Foxn1 nu), immunodeficient SCID (B6.CB17—Prkdc SCID/SzJ), Rag (B6.129S7—Rag1tm1Mom) (lack of B and T cells), athymic (thymectomized neonates and adults C57Bl/6J) and cyclosporine A-treated mice were designed to test the hypothesis that T-cell deficiency and/or lack of a thymus promote scar-less skin repair in mice. Surprisingly, the obtained results revealed that the capability for regenerative healing is a unique feature of nude mice and is not a direct consequence of immunodeficiency. The lack of a thymus and/or T-cells is not sufficient to support scar-less healing [43]. The analysis of post-injured skin tissues collected from Foxn1-deficient mice revealed the lack of a scar, high levels of hyaluronic acid, low levels of collagen and pro-scarring cytokines (PDGF-B and TGF β1) [43] (Scheme 1).

Ensuing studies showed a unique bimodal pattern of Mmp-9 and Mmp-13 expression during skin wound healing in nude mice [42,105]. The first wave of high expression was observed in the epidermis during the inflammatory phase. The secondary induction of Mmps expression was detected in the dermal portion of the skin during the remodelling phase of wound healing. The authors hypothesised that the bimodal pattern of the Mmps expression might be responsible for accelerated remodelling and scar-free healing [106]. Interestingly, this unusual pattern of Mmps regulation was observed for the first time during limb regeneration in amphibians [107,108].

### 4.2. Foxn1 in Reparative (Scar-Forming) Skin Wound Healing

The analysis of post-injured skin tissues of Foxn1-deficient mice has revealed the ability of these mice to heal cutaneous wounds in a regenerative manner. Thus, the focus was shifted towards transcription factor Foxn1 as a possible regulator of the switch between scar-forming and scar-free healing. The next series of studies focused on determining the role of transcriptional factor Foxn1 in the skin injury repair process. The experiments performed on young, adult and old Foxn1::Egfp transgenic and C57BL/6J (B6) mice showed a gradual decrease in *Foxn1* mRNA levels in post-wounded skin tissues during the first seven days after injury [16,21]. Foxn1-positive cells accumulated in the post-wounded skin area. The thickened epidermis at the wound margin and the leading epithelial tongue migrating underneath the scab in the transgenic Foxn1::Egfp mouse model were formed by Foxn1-eGFP-positive cells (Figure 1B). An immunodetection assay revealed that every single cell comprising the leading epidermal tongue showed Foxn1-eGFP and Krt16 co-localisation [16,21]. Wawersik et al. demonstrated that Krt16-positive keratinocytes form the hyperproliferative, activated epithelia in the leading tip during the re-epithelialisation [109], whereas Prowse et al. showed that Foxn1 overexpression in the skin of transgenic mice stimulates Krt6, which is a partner of Krt16 [14]. Moreover, Foxn1 overexpression in the primary culture of keratinocytes triggers Krt6a and Krt6b protein expression [44]. Thus, all those data support the concept that Foxn1 as a transcription activator contribute to the activation of keratins (i.e., Krt16) required for epithelial resurfacing of post-wounded skin tissues [16]. Interestingly, at days 4–6 after injury, Foxn1-eGFP-positive cells were observed not only in the neoepidermis but also in the dermal part of the skin [16]. The majority of Foxn1-eGFP-positive cells, found at the wound margin and in the re-epithelialised epidermis, co-localised with E-cadherin, indicating epidermal cell characteristics [16,21]. However, the flow cytometric analysis of cells isolated from post-wounded day 5 skin tissues of adult Foxn1::Egfp mice revealed the presence not only of eGFP and E-cadherin positive cells but also of Foxn1-eGFP and double E-cadherin/N-cadherin (epithelial/mesenchymal markers)-positive cells [16,21]. The population of Foxn1-eGFP/E-cadherin/N-cadherin-positive cells increased from 3.41% in uninjured skin tissues to 12.5% at day 5 after injury. Moreover, the increase in mRNA expression levels of EMT markers, including *Snail1* and *Mmp-9*, was accompanied by the accumulation of Foxn1-eGFP- and Snail1-positive cells in the dermal portion of post-wounded skin. These results suggest the involvement of Foxn1 in the EMT during the skin wound healing (Scheme 1). Moreover, increased Foxn1 activity at the first post-wounding days indicates that this period is critical for possible intervention targeting switching reparative vs. regenerative skin resolution [16]. A study by Iwano et al. suggests that, during wound healing, the majority of local fibroblasts developed from the epithelium following EMT [110]. The authors imply that the manipulation of cell fate pathways of the epithelium towards re-epithelialisation, while diminishing EMT may serve to prevent excessive fibrosis [110]. 

The initial decrease in *Foxn1* mRNA expression was followed by its increase at post-injured days 14 and 21. Post-wounded skin tissues at days 14-36 displayed Foxn1-eGFP cells not only in the epidermis but also in the papillary dermis [21]. The presence of cells positive for Foxn1/E-cadherin/N-cadherin, along with Foxn1-eGFP/α-smooth muscle actin (αSMA- myofibroblast marker)-positive cells in the papillary dermis, identified Foxn1 as a potential factor controlling the remodelling phase of the skin wound healing process.

Overall, this pioneer study of Foxn1 and skin wound healing implies a regulatory role of Foxn1 in the re-epithelialisation, EMT and remodelling (scar formation) stage of the skin wound healing process [16,21].

### 4.3. Foxn1 as a Transcriptional Switch between Scar-Free and Scar-Forming Skin Healing

**Scheme 2:**
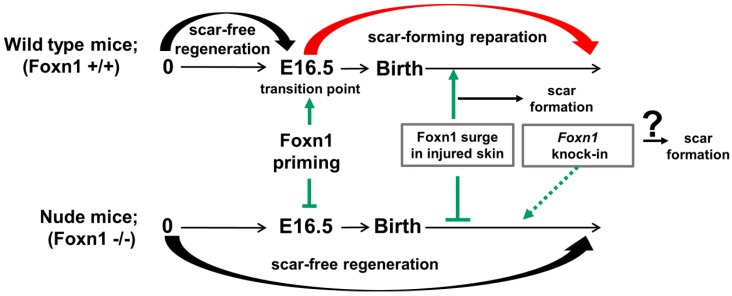
The scheme depicts differences between patterns of skin wound healing resolutions recognised as reparation (scar-forming) or regeneration (scar-free; perfect healing), attributed to Foxn1-active (Foxn1+/+) and Foxn1-deficient (Foxn1−/−; nude) mice, respectively. Regenerative healing of skin wounds occurs until embryonic day 16.5 (E16.5), when Foxn1 is non-active in both models. Perfect healing begins to diminish from E16.5 in Foxn1+/+ mice due to the Foxn1 expression in the epidermis (Foxn1 priming) that correlates with the physiological transition from scar-free to scar-forming healing (E16.5 transition point). The Foxn1 loss-of-function mutation in nude mice maintains skin regenerative abilities throughout adulthood. The effect of conditional Foxn1 knock-in in the skin of adult Foxn1−/− nude mice would answer the question regarding whether “turning on” Foxn1 activity in mature skin could lead to acquiring a reparative (scar-forming) phenotype or whether there is no effect since the skin of nude mice is not responsive to Foxn1 signals due to a lack of Foxn1 priming at E16.5. The answer to the questions await further research.

Scar-less skin wound healing observed in Foxn1-deficient mice showed a strict resemblance to the scar-free healing previously described in the mammalian foetuses (mice, rats, pigs, monkeys, and humans) [39]. The phenomenon of regenerative (scar-free) skin wound healing in developing embryo occurs until the third trimester of gestation [38,111,112]. Thereafter, skin injury is resolved in an adult (scar forming) manner. The mechanisms regulating the transition from regenerative to reparative (scar-forming) healing in foetuses are not completely understood. As discussed above (see Paragraph 2.2.1), the expression of the transcription factor Foxn1 in the epidermis of a mouse embryo begins at embryonic day 16.5, which coincides with the transition point from scar-free to scar-forming skin wound healing (Scheme 2).

The comparison analysis of the transcriptomes from the uninjured skin of E14 and nude mice (models of skin regeneration) with that of E18 and B6 mice (models of skin repair) revealed in nude and E14 skin upregulation of genes, which are normally rapidly translated in response to wounding, associated with tissue remodelling and the immune response [41]. Similar gene categories have been suggested to influence the outcome of injury in other models of regeneration: lower vertebrates [113] and the MRL mouse [114]. The results obtained by Gourevitch et al. showed that the predisposition to immediate post-injured response is also partially true for the MRL mice, which possess an increased population of inflammatory cells during scar-free ear wound closure [114,115]. Collectively, this finding may indicate that a loss of-function mutation in *Foxn1* in nude mice and inactive Foxn1 during the scar-free healing phase in mammalian embryos provide conditions conductive to regeneration [41] (Scheme 2).

### 4.4. Foxn1 among Transcription Factors and Signalling Pathways in the Skin Healing Process

DNA sequencing analysis revealed that Foxn1-deficient skin shows alterations in the expression of other transcriptional factors involved in the skin wound healing, belonging to the Hox and Foxo families [41].

One of the representatives of the Hox family is Hoxb13. Stelnicki et al. identified *HOXB13* as a gene differentially expressed during foetal versus adult wound healing. Similar to *Foxn1* expression, *HOXB13* expression decreased in wounded foetal tissue relative to unwounded foetal controls or wounded adult skin [116]. Experiments performed by Mack et al. showed that the loss of Hoxb13 enhanced the healing process, increased the tensile strength, and reduced the dermal scarring, suggesting that the absence of Hoxb13 creates a more foetal-like environment [117].

Foxo1 and Foxo3 are the representatives of forkhead transcription factors in the O-box sub-family. Foxo transcriptional factors were described as regulators of multiple processes, such as cell cycle arrest, DNA repair [118] cell death [119,120], oxidative stress resistance [121], adipocyte differentiation [122], tumour suppression [123], insulin-regulated gluconeogenesis [124], and wound healing [125]. However, the role of Foxo1 and Foxo3 in skin wound healing remains controversial. Mori et al. demonstrated accelerated and improved quality of skin wound healing in partial knockout Foxo1+/− mice, which exhibit accelerated skin wound healing, decreased scarring, and enhanced keratinocyte migration [7]. Deletion of Foxo3 led to accelerated wound healing in an *in vivo* mouse model [126]. However, there are reports that indicate that deletion of Foxo1 in keratinocytes markedly reduced the rate of wound healing, likely due to the requirement of Foxo1 for keratinocyte transition to a wound-healing phenotype that involves migration and up-regulation of TGF-β1 and its downstream targets, integrin-α3 and -β6 and Mmp-3 and Mmp-9 [127].

Another transcriptional factor implicated in scar-free healing is Msx2. It has been shown that Msx2 is involved in the regeneration of a vertebrate digit organ [128], and its expression differs in the dermis and epidermis between foetal and adult skin [129]. Yeh et al. revealed that Msx2 was transiently upregulated during healing of the excisional skin wounds in wild-type mice [130]. The Msx2 expression coincided with the migratory pattern of epithelial cells. Mice deficient in the homeobox gene Msx2 exhibited faster wound closure with accelerated re-epithelialisation and earlier expression of keratin differentiation markers [130]. Interestingly, Msx2 is expressed together with Foxn1 in the matrix and differentiating cortex of the hair follicles. The deficiency of both transcriptional factors is beneficial for the outcome of the wound healing process [25,130]. Cai et al. suggested that Msx2 and Foxn1 are independently induced to synergistically control the expression of several cortical and cuticle keratins [77,131]. Both transcription factors most likely function in parallel pathways, acting downstream of BMP4 and upstream of Notch1 [77]. Modulating the activity of Msx2 and Foxn1 may involve Wnt signalling, which is reported to act synergistically or antagonistically with the BMP pathway [132].

Wnt signalling is an evolutionally conserved pathway that regulates crucial aspects in embryonic development, homeostasis, regeneration and injury repair [133]. In animals with robust regenerative capacities, the reduction in Wnt decreases their abilities to perform scar-free healing [134]. Kawakami et al. reported that blocking Wnt signalling after amputation of the dorsal fin in zebrafish impaired fin regeneration [135]. Comparison of the transcriptomics profile between nude (model of regeneration) and wild-type (model of reparative healing) mice showed upregulation of the genes involved in Wnt signalling pathways in nude mice [41]. Elevated gene expression in the skin tissues of nude mice was observed for the Wnt ligands *Wisp2*, *Wnt10a* and *Wnt11* and Wnt receptors *frizzled 1* (*Fzd1*) and *frizzled 6* (*Fzd6*). The Wnt mediator *β-catenin* (*Ctnnb1*) was upregulated in the epidermis but not in the skin, whereas downregulation in the skin was observed for the Wnt pathway *inhibitors dickkopf 1* (*Dkk1*), *frizzled related protein* (*Frzb*) and *secreted frizzled-related protein 2* (*Sfrp2*) [41].

In mammalian skin that is characterised by limited regenerative capacities, Wnt signalling is required for the wound healing process. It was described that Wnt activation in epidermal stem cells drives self- renewal and proliferation to promote post-injured wound closure [136] and regulates HFs neogenesis. Stimulation of Wnts in the interfollicular epidermis is crucial for *de novo* forming of HFs. Ito et al. showed that inhibition of Wnt signalling after re-epithelialisation abrogated wounding-induced folliculogenesis [137]. Conversely, studies demonstrated that the canonical Wnt pathway plays a profibrotic role in the post-injured dermis. It has been shown that β-catenin-mediated Tcf-dependent transcription was activated in the proliferative phase of wound healing [138]. Moreover, the canonical Wnt pathway potentially stimulates fibroblasts activation and tissue fibrosis [139].

Experiments performed by Fathke et al. showed the possible involvement of non-canonical Wnt pathway in the skin wound healing process, as they observed transient upregulation of β-catenin-independent Wnts, namely, Wnt4, Wnt5a and Wnt11, in the course of healing [140]. Analysis of mRNA expression levels in post-wounded skin tissues showed increased expression of *Wnt4* at day 7 after injury. The initial decrease in the *Wnt5a and Wnt11* mRNA expression for seven days after injury was followed by a rapid increase at post-injured days 14 and 21 [140]. The mRNA profile of *Wnt5a and Wnt11* genes coincides with the expression of *Foxn1* during the skin wound healing process. Considering that Wnt glycoproteins have been previously shown to regulate Foxn1 expression in the thymus and in hair follicles [59,68], this suggests that Wnt ligands may participate in the regulation of Foxn1 expression during skin wound healing. One of the triggers that activates the endogenous Wnt pathway is hypoxia. Studies performed by Mori et al. demonstrated that Hif-1α modulates the ligand of the non-canonical Wnt pathway, Wnt11, which is induced by hypoxia in many cell types. Elevated endogenous Wnt11 increases the activity of Mmp-2 and Mmp-9 and promotes proliferation, migration and invasion of cancer-derived cells [141]. Similarly, a study by Kur-Piotrowska et al. showed that, in primary cultures of nude keratinocytes transfected with the Ad-Foxn1 construct (exogenous Foxn1) and C57BL/6 keratinocytes (endogenous Foxn1), *Foxn1* expression is induced by hypoxia [44]. Combined, these findings indicate that the induction of Wnt pathway is likely to be caused by hypoxic conditions generated in post-wounded tissues. Nevertheless, the link/interplay between hypoxia, Wnt signalling and Foxn1 action at the injury site continues to await identification. Thus, differences in the Hif-1α expression have been previously detected between reparative healed tissues (adult skin) and privileged for regenerative healing foetal skin. The post-wounded skin of mammalian foetuses (sheep) that heals in a regenerative manner (similar to adult nude mice) showed no Hif-1α expression [142]. In contrast, an increase in Hif-1α expression was detected in post-wounded adult sheep skin that heals in a scar forming, reparative manner [142]. Collectively, the data suggest that, during the cutaneous wound healing process, hypoxia-related genes, including Foxn1, may drive the physiological decision of the type of healing: regeneration or repair.

## 5. Conclusions and Future Directions

In this review, we summarised the findings that have considerably advanced the understanding of the role of Foxn1 in the skin as it has been investigated in various settings, including skin morphogenesis, homeostasis and wound healing (Table 2).

However, many questions remain to obtain a better understanding of the complexity of the Foxn1 function. Specifically, this function refers to differences between Foxn1-primed and Foxn1-non-primed (nude) skin during embryonic development. Does Foxn1 priming develop during neonatal life and lead the skin to acquire characteristic cellular features of skin maturity (e.g., reparative, scar-present skin wound healing) (Scheme 2)? Our recent sequencing data show [41] that the employed skin and epidermis from mouse embryos (E14, model of skin regeneration; E18, model of skin repair) and their adult counterparts, represented by nude (model for skin regeneration) and C57BL/6J wild-type animals (model for skin repair), demonstrated that Foxn1 acts as the transcriptomic signature that determines skin fate. Indeed, the lack of Foxn1 in adult nude, similar to E14 mice, results in an immature, neotenic phenotype of skin that likely drives regenerative skin healing when injury occurs. Notably, Foxn1 action appears to be far greater than that in its host tissue, the epidermis, and it expands to the underlying dermal layer of skin. Current data obtained using dermal fibroblasts derived from the skin of “regenerative” nude mice and their wild-type counterparts revealed multiple differences at molecular and cellular levels between both studied models [47,88]. Additionally, keratinocyte-conditioned media (KCMs) collected from Foxn1-active and Foxn1-deficient mice exhibited the opposite effect on fibroblast cellular features [88]. Overall, these data lead to the conclusion that Foxn1 contributes to skin cell phenotype and behaviour (Scheme 1). This interaction is established early during embryonic development and is continued over the postnatal life. Thus, it appears to be interesting to investigate the effect of the conditional *Foxn1* knock-out in adult skin to answer the question regarding whether turning off Foxn1 activity in mature, Foxn1-primed skin results in the regenerative type of post-injured skin healing. Skin injuries continue to present significant clinical challenges. These include impaired healing, pressure ulcers associated with immobilisation, diabetes, and peripheral vascular disease, scarring, or over-exuberant tissue formation (keloid). Inadequate or incomplete wound healing can lead to impaired immune, mechanical, and physiological function of the skin as well as disfiguring cosmetic defects. Despite many advances during recent years, wound healing remains an area of unmet medical need. Identify novel therapeutic targets in wound healing management will have a tremendous impact on medicine and pharmacy. Since Foxn1 expression is limited to the skin epidermis and thymic epithelium (physiological involution with age), it is highly likely that such topical targeted medical treatments will prove to be both beneficial and safe. Defined Foxn1 action in skin tissues (regeneration, scar healing, non-healing wounds) can stimulate development of new pharmacological treatments to minimise scarring and diminish scar-related morbidity and/or redirect skin repair to regeneration outcome and non-healing wounds to scar-forming. However, understanding of the molecular regulation that allow the body to re-direct healing pathways from scar-forming to scar-free or from non-healing wounds to scar forming remains to be determined.

Another issue that merits further consideration is the regulatory function of Foxn1 in epidermal stem cell activity and behaviour. A variety of studies have documented that daily turnover of interfollicular epidermis as well as HF cyclical bouts of growth both require constant use of stem cells [143,144,145,146]. Within the epidermis, stem cells are placed in the basal layer of keratinocytes, where they exhibit unlimited self-renewal capacity and generate daughter cells that undergo terminal differentiation. Moreover, upon injury, stem cells immediately mobilise from their niche to administer rapid tissue repair [147]. Interestingly, this characteristic pattern of stem cells activity manifested under steady-state conditions as well as during wounding appears to be in concert with the gradient of the Foxn1 expression in the adequate/parallel physiological events occurring in the skin [16]. Furthermore, consistent with previous reports [13,14,19,20], the dynamics of the Foxn1 expression required for proper keratinocyte terminal differentiation and necessary for HFs morphogenesis are likely to overlap the changes in stem cell activity that are serving as the strategy to control skin homeostasis. Thus, although there is a lack of solid experimental evidence to support the connection between the Foxn1 expression and stem cells’ fate, existing data allow one to conclude that Foxn1 might be a necessary cue for the proper function of stem cells in the skin. In this regard, the Wnt pathway might be identified as the link between Foxn1 and stem cells since both showed individual input in the maintenance of skin homeostasis [59].

Underlined in this review the key role of Foxn1 in all aspects of skin biology: development, homeostasis and wound healing, together with the complexity of its action require further research with particular attention in determining what are the downstream genes targeted by Foxn1.

## Figures and Tables

**Table 1:** Characterisation of Foxn1/FOXN1 mutation in mouse (nude) and human (Nude/SCID).

Characteristic of Foxn1 Deficiency	
Mouse (Nude)	Human (Nude/SCID)
Mutations in the *Foxn1* gene identified in the mouse allele *nu*, located on chromosomes 11; a single base-pair deletion in the *Foxn1* coding sequence leads to a frame shift and premature termination in the DBD [22,67];	Mutation in human *FOXN1* gene recognised as a homozygous C-to-T transition (CGA to TGA) at nucleotide position 792 resulted in a nonsense mutation at residue 255 (R255X) in exon 5 [70];
The translated FOXN1, Foxn1, proteins are nonfunctional in human and mice, respectively and lead to similar defects [23,24,25]; Pleiotropic mutation categorised into two independent phenotypic responses: (i) impaired skin keratinisation and aberrant HFs development [13,19,23] and (ii) thymus dysgenesis that leads to T-cell deficiency [23,67];	
Mutants represent multiple skin defects [13,19]: (i) abnormalities in the HF structure manifested by short and bent hair shafts with a lack of cuticles in IRS and ORS and visible abnormal globular aggregates in the hair cortex. Aberrant hair rarely protrudes from the follicles; hence, nude mice fail to grow visible hair; (ii) disturbances of keratinisation reflected by a reduced number of tonofilaments in the basal, spinous and granular layers and irregularly arranged lamellae of corneocytes in the stratum corneum [13]; (iii) nails malfunctions (severe onychodystrophy and brachyonychia) reflected by blunt, broken and irregularly ended nails; a thinned nail plate; an altered nail matrix; and loss of Krt1 expression and aberrant expression of Flg [71]; Nude phenotype is associated with loss of the expression of the acidic hair keratin 3 gene (*mHA3*) in HFs and *mHb5* is skin, both transcripts are direct Foxn1 targets [33,34].	Foetuses at 15th weeks show tight, shiny and smooth skin associated with a lack of thymus, anencephaly and spina bifida, indicating that beside its role in the thymus and skin epithelium, FOXN1 might also be involved in neurulation in humans [72]; Infant mutants demonstrate complete alopecia of the scalp, eyebrow, and eyelashes and nail dystrophy associated with a primary severe T-cell immunodeficiency [23,73].

**Table 2:** Characteristic features of skin physiology attributed to Foxn1 activity or Foxn1 deficiency.

	Attributes to Foxn1 Activity in the Mice Skin	Attributes to Foxn1 Deficiency in the Mice Skin
Development	*Prenatal stage:* Foxn1 expression detected on E13 in the developing nasal region; and by E15.5–16.5 Foxn1 occupied entire epidermis including developing HF of the fur coat [19]; 2065 genes differentially regulated (up and down) between skin from B6 embryo at E14 (model of skin regeneration) and E18 (model of skin reparation) [41]; *Early neonatal stage:* Foxn1 expression (postpartum days P1–P2) reported in the hair shaft and throughout the developing IRS and in a matrix compartment of hair bulb suggesting that *Foxn1* correlates with the onset of terminal differentiation [19];	*Prenatal stage*: Lack of Foxn1 activity at E16.5 (Foxn1 priming) keeps skin of nude mice in the immature stage resembling the phenomena of neoteny [41];
Homeostasis	Foxn1 expression in epithelial cells (epidermis) initiates terminal differentiation program and stimulates neighboring epithelial cell proliferation in paracrine manner [14,19,51]; Foxn1 participates in the development of skin epithelial cells coloration through activation of epithelial cells to emit signals (FGF-2) recognisable by melanocytes [54]; Epidermal Foxn1 expression impact the skin: (i) decreased levels of anti-fibrotic cytokine Tgfbeta3; (ii) cultured DF exhibited reduced adipogenic differentiation capacity [88];	Impaired keratinisation of epidermis and hair shaft results in “hairless phenotype” [24,25]; Aberrant differentiation of epidermal keratinocytes manifests by inhibition of early differentiation markers (Krt1, -10), overproduction and accumulation of late differentiation markers (profilaggrin, loricrin, involucrin) and abnormal thickening of epidermis [13,14]; Increased amounts of cholesterol sulfate, phospholipids, sphingolipids and fatty acids in the skin when compare to the Foxn1 -active mouse models [89];
Wound healing	The process of healing occurs with fibrosis and scar formation, a condition characterised by excessive deposition of ECM protein rich in collagens [97,98,99]; Foxn1 acts as a key molecular player in re-epithelialisation and EMT processes in post-wounded skin tissues [16].	Perfect healing in the process of regeneration characterised by lack of a scar, high levels of hyaluronic acid, low levels of collagen and pro-scarring cytokines (PDGF-B and TGF β1), and unique bimodal pattern of Mmp-9 and Mmp-13 expression [42,43].

## References

[1] Slominski A.T., Manna P.R., Tuckey R.C. (2015). On the role of skin in the regulation of local and systemic steroidogenic activities. Steroids.

[2] Slominski A.T., Zmijewski M.A., Skobowiat C., Zbytek B., Slominski R.M., Steketee J.D. (2012). Sensing the environment: Regulation of local and global homeostasis by the skin’s neuroendocrine system. Adv. Anat. Embryol. Cell. Biol..

[3] Werner S., Krieg T., Smola H. (2007). Keratinocyte-fibroblast interactions in wound healing. J. Investig. Dermatol..

[4] Bellavia G., Fasanaro P., Melchionna R., Capogrossi M.C., Napolitano M. (2014). Transcriptional control of skin reepithelialization. J. Dermatol. Sci..

[5] Teng A., Nair M., Wells J., Segre J.A., Dai X. (2007). Strain-dependent perinatal lethality of Ovol1-deficient mice and identification of Ovol2 as a downstream target of Ovol1 in skin epidermis. Biochim. Biophys. Acta.

[6] Lee B., Villarreal-Ponce A., Fallahi M., Ovadia J., Sun P., Yu Q.C., Ito S., Sinha S., Nie Q., Dai X. (2014). Transcriptional mechanisms link epithelial plasticity to adhesion and differentiation of epidermal progenitor cells. Dev. Cell.

[7] Mori R., Tanaka K., de Kerckhove M., Okamoto M., Kashiyama K., Kim S., Kawata T., Komatsu T., Park S., Ikematsu K. (2014). Reduced FOXO1 expression accelerates skin wound healing and attenuates scarring. Am. J. Pathol..

[8] Donati G., Proserpio V., Lichtenberger B.M., Natsuga K., Sinclair R., Fujiwara H., Watt F.M. (2014). Epidermal Wnt/beta-catenin signaling regulates adipocyte differentiation via secretion of adipogenic factors. Proc. Natl. Acad. Sci. USA.

[9] Schafer M., Werner S. (2007). Transcriptional control of wound repair. Annu. Rev. Cell Dev. Biol..

[10] Lichtenberger B.M., Mastrogiannaki M., Watt F.M. (2016). Epidermal beta-catenin activation remodels the dermis via paracrine signalling to distinct fibroblast lineages. Nat. Commun..

[11] Corbeaux T., Hess I., Swann J.B., Kanzler B., Haas-Assenbaum A., Boehm T. (2010). Thymopoiesis in mice depends on a Foxn1-positive thymic epithelial cell lineage. Proc. Natl. Acad. Sci. USA.

[12] Boehm T., Swann J.B. (2013). Thymus involution and regeneration: Two sides of the same coin?. Nat. Rev. Immunol..

[13] Brissette J.L., Li J., Kamimura J., Lee D., Dotto G.P. (1996). The product of the mouse nude locus, WHN, regulates the balance between epithelial cell growth and differentiation. Genes Dev..

[14] Prowse D.M., Lee D., Weiner L., Jiang N., Magro C.M., Baden H.P., Brissette J.L. (1999). Ectopic expression of the nude gene induces hyperproliferation and defects in differentiation: Implications for the self-renewal of cutaneous epithelia. Dev. Biol..

[15] Schlake T. (2001). The nude gene and the skin. Exp. Dermatol..

[16] Gawronska-Kozak B., Grabowska A., Kur-Piotrowska A., Kopcewicz M. (2016). Foxn1 transcription factor regulates wound healing of skin through promoting epithelial-mesenchymal transition. PLoS ONE.

[17] Bredenkamp N., Nowell C.S., Blackburn C.C. (2014). Regeneration of the aged thymus by a single transcription factor. Development.

[18] Terszowski G., Muller S.M., Bleul C.C., Blum C., Schirmbeck R., Reimann J., Pasquier L.D., Amagai T., Boehm T., Rodewald H.R. (2006). Evidence for a functional second thymus in mice. Science.

[19] Lee D., Prowse D.M., Brissette J.L. (1999). Association between mouse nude gene expression and the initiation of epithelial terminal differentiation. Dev. Biol..

[20] Baxter R.M., Brissette J.L. (2002). Role of the nude gene in epithelial terminal differentiation. J. Investig. Dermatol..

[21] Kopcewicz M.M., Kur-Piotrowska A., Bukowska J., Gimble J.M., Gawronska-Kozak B. (2017). Foxn1 and mmp-9 expression in intact skin and during excisional wound repair in young, adult, and old c57bl/6 mice. Wound Repair Regen..

[22] Nehls M., Pfeifer D., Schorpp M., Hedrich H., Boehm T. (1994). New member of the winged-helix protein family disrupted in mouse and rat nude mutations. Nature.

[23] Pignata C., Fiore M., Guzzetta V., Castaldo A., Sebastio G., Porta F., Guarino A. (1996). Congenital alopecia and nail dystrophy associated with severe functional t-cell immunodeficiency in two sibs. Am. J. Med. Genet..

[24] Flanagan S.P. (1966). ‘Nude’, a new hairless gene with pleiotropic effects in the mouse. Genet. Res..

[25] Mecklenburg L., Tychsen B., Paus R. (2005). Learning from nudity: Lessons from the nude phenotype. Exp. Dermatol..

[26] Barbul A., Shawe T., Rotter S.M., Efron J.E., Wasserkrug H.L., Badawy S.B. (1989). Wound healing in nude mice: A study on the regulatory role of lymphocytes in fibroplasia. Surgery.

[27] Schlake T., Schorpp M., Boehm T. (2000). Formation of regulator/target gene relationships during evolution. Gene.

[28] Schorpp M., Hofmann M., Dear T.N., Boehm T. (1997). Characterization of mouse and human nude genes. Immunogenetics.

[29] Schlake T., Schorpp M., Nehls M., Boehm T. (1997). The nude gene encodes a sequence-specific DNA binding protein with homologs in organisms that lack an anticipatory immune system. Proc. Natl. Acad. Sci. USA.

[30] Clark K.L., Halay E.D., Lai E., Burley S.K. (1993). Co-crystal structure of the hnf-3/fork head DNA-recognition motif resembles histone h5. Nature.

[31] Tsai K.L., Huang C.Y., Chang C.H., Sun Y.J., Chuang W.J., Hsiao C.D. (2006). Crystal structure of the human foxk1a-DNA complex and its implications on the diverse binding specificity of winged helix/forkhead proteins. J. Biol. Chem..

[32] Nakagawa S., Gisselbrecht S.S., Rogers J.M., Hartl D.L., Bulyk M.L. (2013). DNA-binding specificity changes in the evolution of forkhead transcription factors. Proc. Natl. Acad. Sci. USA.

[33] Meier N., Dear T.N., Boehm T. (1999). Whn and mha3 are components of the genetic hierarchy controlling hair follicle differentiation. Mech. Dev..

[34] Schlake T., Schorpp M., Maul-Pavicic A., Malashenko A.M., Boehm T. (2000). Forkhead/winged-helix transcription factor whn regulates hair keratin gene expression: Molecular analysis of the nude skin phenotype. Dev. Dyn..

[35] Calautti E., Li J., Saoncella S., Brissette J.L., Goetinck P.F. (2005). Phosphoinositide 3-kinase signaling to akt promotes keratinocyte differentiation versus death. J. Biol. Chem..

[36] Li J., Baxter R.M., Weiner L., Goetinck P.F., Calautti E., Brissette J.L. (2007). Foxn1 promotes keratinocyte differentiation by regulating the activity of protein kinase C. Differentiation.

[37] Nehls M., Kyewski B., Messerle M., Waldschutz R., Schuddekopf K., Smith A.J., Boehm T. (1996). Two genetically separable steps in the differentiation of thymic epithelium. Science.

[38] Ihara S., Motobayashi Y., Nagao E., Kistler A. (1990). Ontogenetic transition of wound healing pattern in rat skin occurring at the fetal stage. Development.

[39] Colwell A.S., Longaker M.T., Lorenz H.P. (2005). Mammalian fetal organ regeneration. Adv. Biochem. Eng. Biotechnol..

[40] Colwell A.S., Longaker M.T., Peter Lorenz H. (2008). Identification of differentially regulated genes in fetal wounds during regenerative repair. Wound Repair Regen..

[41] Kur-Piotrowska A., Kopcewicz M., Kozak L.P., Sachadyn P., Grabowska A., Gawronska-Kozak B. (2017). Neotenic phenomenon in gene expression in the skin of Foxn1- deficient (nude) mice—A projection for regenerative skin wound healing. BMC Genom..

[42] Gawronska-Kozak B. (2011). Scarless skin wound healing in Foxn1 deficient (nude) mice is associated with distinctive matrix metalloproteinase expression. Matrix Biol..

[43] Gawronska-Kozak B., Bogacki M., Rim J.S., Monroe W.T., Manuel J.A. (2006). Scarless skin repair in immunodeficient mice. Wound Repair Regen..

[44] Kur-Piotrowska A., Bukowska J., Kopcewicz M.M., Dietrich M., Nynca J., Slowinska M., Gawronska-Kozak B. (2018). Foxn1 expression in keratinocytes is stimulated by hypoxia: Further evidence of its role in skin wound healing. Sci. Rep..

[45] Janes S.M., Ofstad T.A., Campbell D.H., Watt F.M., Prowse D.M. (2004). Transient activation of FOXN1 in keratinocytes induces a transcriptional programme that promotes terminal differentiation: Contrasting roles of FOXN1 and Akt. J. Cell Sci..

[46] Janes S.M., Ofstad T.A., Campbell D.H., Eddaoudi A., Warnes G., Davies D., Watt F.M. (2009). Pi3-kinase-dependent activation of apoptotic machinery occurs on commitment of epidermal keratinocytes to terminal differentiation. Cell Res..

[47] Gawronska-Kozak B., Kirk-Ballard H. (2013). Cyclosporin a reduces matrix metalloproteinases and collagen expression in dermal fibroblasts from regenerative Foxn1 deficient (nude) mice. Fibrog. Tissue Repair.

[48] Dai J., Brooks Y., Lefort K., Getsios S., Dotto G.P. (2013). The retinoid-related orphan receptor roralpha promotes keratinocyte differentiation via Foxn1. PLoS ONE.

[49] Fuchs E. (2007). Scratching the surface of skin development. Nature.

[50] Alonso L., Fuchs E. (2006). The hair cycle. J. Cell Sci..

[51] Schmidt-Ullrich R., Paus R. (2005). Molecular principles of hair follicle induction and morphogenesis. Bioessays.

[52] Legue E., Nicolas J.F. (2005). Hair follicle renewal: Organization of stem cells in the matrix and the role of stereotyped lineages and behaviors. Development.

[53] Dai X., Segre J.A. (2004). Transcriptional control of epidermal specification and differentiation. Curr. Opin. Genet. Dev..

[54] Weiner L., Han R., Scicchitano B.M., Li J., Hasegawa K., Grossi M., Lee D., Brissette J.L. (2007). Dedicated epithelial recipient cells determine pigmentation patterns. Cell.

[55] Yuan S., Li F., Meng Q., Zhao Y., Chen L., Zhang H., Xue L., Zhang X., Lengner C., Yu Z. (2015). Post-transcriptional regulation of keratinocyte progenitor cell expansion, differentiation and hair follicle regression by mir-22. PLoS Genet..

[56] DasGupta R., Fuchs E. (1999). Multiple roles for activated LEF/TCF transcription complexes during hair follicle development and differentiation. Development.

[57] Huelsken J., Vogel R., Erdmann B., Cotsarelis G., Birchmeier W. (2001). Beta-catenin controls hair follicle morphogenesis and stem cell differentiation in the skin. Cell.

[58] Zhang Y., Andl T., Yang S.H., Teta M., Liu F., Seykora J.T., Tobias J.W., Piccolo S., Schmidt-Ullrich R., Nagy A. (2008). Activation of beta-catenin signaling programs embryonic epidermis to hair follicle fate. Development.

[59] Hu B., Lefort K., Qiu W., Nguyen B.C., Rajaram R.D., Castillo E., He F., Chen Y., Angel P., Brisken C. (2010). Control of hair follicle cell fate by underlying mesenchyme through a CSL-Wnt5a-Foxn1 regulatory axis. Genes Dev..

[60] Nakamura Y., Ichinohe M., Hirata M., Matsuura H., Fujiwara T., Igarashi T., Nakahara M., Yamaguchi H., Yasugi S., Takenawa T. (2008). Phospholipase c-delta1 is an essential molecule downstream of Foxn1, the gene responsible for the nude mutation, in normal hair development. FASEB J..

[61] Johns S.A., Soullier S., Rashbass P., Cunliffe V.T. (2005). Foxn1 is required for tissue assembly and desmosomal cadherin expression in the hair shaft. Dev. Dyn..

[62] Hirobe T. (2014). Keratinocytes regulate the function of melanocytes. Dermatol. Sin..

[63] Pla P., Solov’eva O., Moore R., Alberti C., Kunisada T., Larue L. (2004). *Dct*::Lacz es cells: A novel cellular model to study melanocyte determination and differentiation. Pigment Cell Res..

[64] Van Den Bossche K., Naeyaert J.M., Lambert J. (2006). The quest for the mechanism of melanin transfer. Traffic.

[65] Cardinali G., Ceccarelli S., Kovacs D., Aspite N., Lotti L.V., Torrisi M.R., Picardo M. (2005). Keratinocyte growth factor promotes melanosome transfer to keratinocytes. J. Investig. Dermatol..

[66] Wu X., Hammer J.A. (2014). Melanosome transfer: It is best to give and receive. Curr. Opin. Cell Biol..

[67] Segre J.A., Nemhauser J.L., Taylor B.A., Nadeau J.H., Lander E.S. (1995). Positional cloning of the nude locus: Genetic, physical, and transcription maps of the region and mutations in the mouse and rat. Genomics.

[68] Balciunaite G., Keller M.P., Balciunaite E., Piali L., Zuklys S., Mathieu Y.D., Gill J., Boyd R., Sussman D.J., Hollander G.A. (2002). Wnt glycoproteins regulate the expression of Foxn1, the gene defective in nude mice. Nat. Immunol..

[69] Eaton G.J. (1976). Hair growth cycles and wave patterns in “nude” mice. Transplantation.

[70] Frank J., Pignata C., Panteleyev A.A., Prowse D.M., Baden H., Weiner L., Gaetaniello L., Ahmad W., Pozzi N., Cserhalmi-Friedman P.B. (1999). Exposing the human nude phenotype. Nature.

[71] Mecklenburg L., Paus R., Halata Z., Bechtold L.S., Fleckman P., Sundberg J.P. (2004). Foxn1 is critical for onycholemmal terminal differentiation in nude (Foxn1) mice. J. Investig. Dermatol..

[72] Amorosi S., D’Armiento M., Calcagno G., Russo I., Adriani M., Christiano A.M., Weiner L., Brissette J.L., Pignata C. (2008). Foxn1 homozygous mutation associated with anencephaly and severe neural tube defect in human athymic nude/scid fetus. Clin. Genet..

[73] Radha Rama Devi A., Panday N.N., Naushad S.M. (2017). Foxn1 Italian founder mutation in Indian family: Implications in prenatal diagnosis. Gene.

[74] Potter C.S., Pruett N.D., Kern M.J., Baybo M.A., Godwin A.R., Potter K.A., Peterson R.L., Sundberg J.P., Awgulewitsch A. (2011). The nude mutant gene Foxn1 is a hoxc13 regulatory target during hair follicle and nail differentiation. J. Investig. Dermatol..

[75] Mangan S., Alon U. (2003). Structure and function of the feed-forward loop network motif. Proc. Natl. Acad. Sci. USA.

[76] Bohr S., Patel S.J., Vasko R., Shen K., Huang G., Yarmush M.L., Berthiaume F. (2013). Highly upregulated lhx2 in the Foxn1−/− nude mouse phenotype reflects a dysregulated and expanded epidermal stem cell niche. PLoS ONE.

[77] Cai J., Lee J., Kopan R., Ma L. (2009). Genetic interplays between Msx2 and Foxn1 are required for notch1 expression and hair shaft differentiation. Dev. Biol..

[78] Vigliano I., Gorrese M., Fusco A., Vitiello L., Amorosi S., Panico L., Ursini M.V., Calcagno G., Racioppi L., Del Vecchio L. (2011). Foxn1 mutation abrogates prenatal t-cell development in humans. J. Med. Genet..

[79] Coolen N.A., Schouten K.C., Middelkoop E., Ulrich M.M. (2010). Comparison between human fetal and adult skin. Arch. Dermatol. Res..

[80] Quigley D.A., Kandyba E., Huang P., Halliwill K.D., Sjolund J., Pelorosso F., Wong C.E., Hirst G.L., Wu D., Delrosario R. (2016). Gene expression architecture of mouse dorsal and tail skin reveals functional differences in inflammation and cancer. Cell Rep..

[81] Chen L., Mirza R., Kwon Y., DiPietro L.A., Koh T.J. (2015). The murine excisional wound model: Contraction revisited. Wound Repair Regen..

[82] Adriani M., Martinez-Mir A., Fusco F., Busiello R., Frank J., Telese S., Matrecano E., Ursini M.V., Christiano A.M., Pignata C. (2004). Ancestral founder mutation of the nude (Foxn1) gene in congenital severe combined immunodeficiency associated with alopecia in southern Italy population. Ann. Hum. Genet..

[83] Ruan L., Zhang Z., Mu L., Burnley P., Wang L., Coder B., Zhuge Q., Su D.M. (2014). Biological significance of Foxn1 gain-of-function mutations during t and b lymphopoiesis in juvenile mice. Cell Death Dis..

[84] Kong W., Li S., Liu C., Bari A.S., Longaker M.T., Lorenz H.P. (2006). Epithelial-mesenchymal transition occurs after epidermal development in mouse skin. Exp. Cell Res..

[85] Wondimu A., Weir L., Robertson D., Mezentsev A., Kalachikov S., Panteleyev A.A. (2012). Loss of ARNT (Hif1beta) in mouse epidermis triggers dermal angiogenesis, blood vessel dilation and clotting defects. Lab. Investig..

[86] El Ghalbzouri A., Ponec M. (2004). Diffusible factors released by fibroblasts support epidermal morphogenesis and deposition of basement membrane components. Wound Repair Regen..

[87] Maas-Szabowski N., Shimotoyodome A., Fusenig N.E. (1999). Keratinocyte growth regulation in fibroblast cocultures via a double paracrine mechanism. J. Cell Sci..

[88] Bukowska J., Kopcewicz M., Kur-Piotrowska A., Szostek-Mioduchowska A.Z., Walendzik K., Gawronska-Kozak B. (2018). Effect of TGFbeta1, TGFbeta3 and keratinocyte conditioned media on functional characteristics of dermal fibroblasts derived from reparative (Balb/c) and regenerative (Foxn1 deficient; nude) mouse models. Cell Tissue Res..

[89] Lanzini J., Dargere D., Regazzetti A., Tebani A., Laprevote O., Auzeil N. (2015). Changing in lipid profile induced by the mutation of Foxn1 gene: A lipidomic analysis of nude mice skin. Biochimie.

[90] Stemmer K., Kotzbeck P., Zani F., Bauer M., Neff C., Muller T.D., Pfluger P.T., Seeley R.J., Divanovic S. (2015). Thermoneutral housing is a critical factor for immune function and diet-induced obesity in c57bl/6 nude mice. Int. J. Obes..

[91] Hirata M., Suzuki M., Ishii R., Satow R., Uchida T., Kitazumi T., Sasaki T., Kitamura T., Yamaguchi H., Nakamura Y. (2011). Genetic defect in phospholipase cdelta1 protects mice from obesity by regulating thermogenesis and adipogenesis. Diabetes.

[92] Schmidt B.A., Horsley V. (2013). Intradermal adipocytes mediate fibroblast recruitment during skin wound healing. Development.

[93] Rivera-Gonzalez G., Shook B., Horsley V. (2014). Adipocytes in skin health and disease. Cold Spring Harb. Perspect. Med..

[94] Alexander C.M., Kasza I., Yen C.L., Reeder S.B., Hernando D., Gallo R.L., Jahoda C.A., Horsley V., MacDougald O.A. (2015). Dermal white adipose tissue: A new component of the thermogenic response. J. Lipid Res..

[95] Kruglikov I.L., Scherer P.E. (2016). Dermal adipocytes: From irrelevance to metabolic targets?. Trends Endocrinol. Metab..

[96] Mastrogiannaki M., Lichtenberger B.M., Reimer A., Collins C.A., Driskell R.R., Watt F.M. (2016). Beta-catenin stabilization in skin fibroblasts causes fibrotic lesions by preventing adipocyte differentiation of the reticular dermis. J. Investig. Dermatol..

[97] Werner S., Grose R. (2003). Regulation of wound healing by growth factors and cytokines. Physiol. Rev..

[98] Singer A.J., Clark R.A. (1999). Cutaneous wound healing. N. Engl. J. Med..

[99] Gurtner G.C., Werner S., Barrandon Y., Longaker M.T. (2008). Wound repair and regeneration. Nature.

[100] Gill S.E., Parks W.C. (2008). Metalloproteinases and their inhibitors: Regulators of wound healing. Int. J. Biochem. Cell Biol..

[101] Clark L.D., Clark R.K., Heber-Katz E. (1998). A new murine model for mammalian wound repair and regeneration. Clin. Immunol. Immunopathol..

[102] Goss R.J., Grimes L.N. (1975). Epidermal downgrowths in regenerating rabbit ear holes. J. Morphol..

[103] Heydemann A. (2012). The super super-healing MRL mouse strain. Front. Biol..

[104] Gawronska-Kozak B. (2004). Regeneration in the ears of immunodeficient mice: Identification and lineage analysis of mesenchymal stem cells. Tissue Eng..

[105] Manuel J.A., Gawronska-Kozak B. (2006). Matrix metalloproteinase 9 (MMP-9) is upregulated during scarless wound healing in athymic nude mice. Matrix Biol..

[106] Gawronska-Kozak B., Grabowska A., Kopcewicz M., Kur A. (2014). Animal models of skin regeneration. Reprod. Biol..

[107] Yang E.V., Gardiner D.M., Carlson M.R., Nugas C.A., Bryant S.V. (1999). Expression of MMP-9 and related matrix metalloproteinase genes during axolotl limb regeneration. Dev. Dyn..

[108] Kato T., Miyazaki K., Shimizu-Nishikawa K., Koshiba K., Obara M., Mishima H.K., Yoshizato K. (2003). Unique expression patterns of matrix metalloproteinases in regenerating newt limbs. Dev. Dyn..

[109] Wawersik M., Coulombe P.A. (2000). Forced expression of keratin 16 alters the adhesion, differentiation, and migration of mouse skin keratinocytes. Mol. Biol. Cell.

[110] Iwano M., Plieth D., Danoff T.M., Xue C., Okada H., Neilson E.G. (2002). Evidence that fibroblasts derive from epithelium during tissue fibrosis. J. Clin. Investig..

[111] Longaker M.T., Whitby D.J., Adzick N.S., Crombleholme T.M., Langer J.C., Duncan B.W., Bradley S.M., Stern R., Ferguson M.W., Harrison M.R. (1990). Studies in fetal wound healing, vi. Second and early third trimester fetal wounds demonstrate rapid collagen deposition without scar formation. J. Pediatr. Surg..

[112] Lorenz H.P., Adzick N.S. (1993). Scarless skin wound repair in the fetus. West. J. Med..

[113] Sousounis K., Michel C.S., Bruckskotten M., Maki N., Borchardt T., Braun T., Looso M., Tsonis P.A. (2013). A microarray analysis of gene expression patterns during early phases of newt lens regeneration. Mol. Vis..

[114] Gourevitch D., Kossenkov A.V., Zhang Y., Clark L., Chang C., Showe L.C., Heber-Katz E. (2014). Inflammation and its correlates in regenerative wound healing: An alternate perspective. Adv. Wound Care.

[115] Cheng C.H., Leferovich J., Zhang X.M., Bedelbaeva K., Gourevitch D., Hatcher C.J., Basson C.T., Heber-Katz E., Marx K.A. (2013). Keratin gene expression profiles after digit amputation in c57bl/6 vs. Regenerative mrl mice imply an early regenerative keratinocyte activated-like state. Physiol. Genom..

[116] Stelnicki E.J., Arbeit J., Cass D.L., Saner C., Harrison M., Largman C. (1998). Modulation of the human homeobox genes prx-2 and hoxb13 in scarless fetal wounds. J. Investig. Dermatol..

[117] Mack J.A., Abramson S.R., Ben Y., Coffin J.C., Rothrock J.K., Maytin E.V., Hascall V.C., Largman C., Stelnicki E.J. (2003). Hoxb13 knockout adult skin exhibits high levels of hyaluronan and enhanced wound healing. FASEB J..

[118] Tran H., Brunet A., Grenier J.M., Datta S.R., Fornace A.J., DiStefano P.S., Chiang L.W., Greenberg M.E. (2002). DNA repair pathway stimulated by the forkhead transcription factor FOXO3a through the gadd45 protein. Science.

[119] Brunet A., Bonni A., Zigmond M.J., Lin M.Z., Juo P., Hu L.S., Anderson M.J., Arden K.C., Blenis J., Greenberg M.E. (1999). Akt promotes cell survival by phosphorylating and inhibiting a forkhead transcription factor. Cell.

[120] Brunet A., Park J., Tran H., Hu L.S., Hemmings B.A., Greenberg M.E. (2001). Protein kinase SGK mediates survival signals by phosphorylating the forkhead transcription factor FKHRL1 (FOXO3a). Mol. Cell Biol..

[121] Van der Horst A., Burgering B.M. (2007). Stressing the role of FOXO proteins in lifespan and disease. Nat. Rev. Mol. Cell Biol..

[122] Nakae J., Kitamura T., Kitamura Y., Biggs W.H., Arden K.C., Accili D. (2003). The forkhead transcription factor FOXO1 regulates adipocyte differentiation. Dev. Cell.

[123] Ramaswamy S., Nakamura N., Sansal I., Bergeron L., Sellers W.R. (2002). A novel mechanism of gene regulation and tumor suppression by the transcription factor FKHR. Cancer Cell.

[124] Puigserver P., Rhee J., Donovan J., Walkey C.J., Yoon J.C., Oriente F., Kitamura Y., Altomonte J., Dong H., Accili D. (2003). Insulin-regulated hepatic gluconeogenesis through FOXO1-pgc-1alpha interaction. Nature.

[125] Tia N., Singh A.K., Pandey P., Azad C.S., Chaudhary P., Gambhir I.S. (2018). Role of forkhead box o (FOXO) transcription factor in aging and diseases. Gene.

[126] Roupe K.M., Veerla S., Olson J., Stone E.L., Sorensen O.E., Hedrick S.M., Nizet V. (2014). Transcription factor binding site analysis identifies FOXO transcription factors as regulators of the cutaneous wound healing process. PLoS ONE.

[127] Ponugoti B., Xu F., Zhang C., Tian C., Pacios S., Graves D.T. (2013). Foxo1 promotes wound healing through the up-regulation of tgf-beta1 and prevention of oxidative stress. J. Cell Biol..

[128] Reginelli A.D., Wang Y.Q., Sassoon D., Muneoka K. (1995). Digit tip regeneration correlates with regions of msx1 (hox 7) expression in fetal and newborn mice. Development.

[129] Stelnicki E.J., Komuves L.G., Holmes D., Clavin W., Harrison M.R., Adzick N.S., Largman C. (1997). The human homeobox genes MSX-1, MSX-2, and MOX-1 are differentially expressed in the dermis and epidermis in fetal and adult skin. Differentiation.

[130] Yeh J., Green L.M., Jiang T.X., Plikus M., Huang E., Chang R.N., Hughes M.W., Chuong C.M., Tuan T.L. (2009). Accelerated closure of skin wounds in mice deficient in the homeobox gene MSX2. Wound Repair Regen..

[131] Ma L., Liu J., Wu T., Plikus M., Jiang T.X., Bi Q., Liu Y.H., Muller-Rover S., Peters H., Sundberg J.P. (2003). ‘Cyclic alopecia’ in MSX2 mutants: Defects in hair cycling and hair shaft differentiation. Development.

[132] Itasaki N., Hoppler S. (2010). Crosstalk between Wnt and bone morphogenic protein signaling: A turbulent relationship. Dev. Dyn..

[133] Bastakoty D., Young P.P. (2016). Wnt/beta-catenin pathway in tissue injury: Roles in pathology and therapeutic opportunities for regeneration. FASEB J..

[134] Whyte J.L., Smith A.A., Helms J.A. (2012). Wnt signaling and injury repair. Cold Spring Harb. Perspect. Biol..

[135] Kawakami Y., Rodriguez Esteban C., Raya M., Kawakami H., Marti M., Dubova I., Izpisua Belmonte J.C. (2006). Wnt/beta-catenin signaling regulates vertebrate limb regeneration. Genes Dev..

[136] Lim X., Tan S.H., Koh W.L., Chau R.M., Yan K.S., Kuo C.J., van Amerongen R., Klein A.M., Nusse R. (2013). Interfollicular epidermal stem cells self-renew via autocrine Wnt signaling. Science.

[137] Ito M., Yang Z., Andl T., Cui C., Kim N., Millar S.E., Cotsarelis G. (2007). Wnt-dependent de novo hair follicle regeneration in adult mouse skin after wounding. Nature.

[138] Cheon S.S., Cheah A.Y., Turley S., Nadesan P., Poon R., Clevers H., Alman B.A. (2002). Beta-catenin stabilization dysregulates mesenchymal cell proliferation, motility, and invasiveness and causes aggressive fibromatosis and hyperplastic cutaneous wounds. Proc. Natl. Acad. Sci. USA.

[139] Akhmetshina A., Palumbo K., Dees C., Bergmann C., Venalis P., Zerr P., Horn A., Kireva T., Beyer C., Zwerina J. (2012). Activation of canonical Wnt signalling is required for TGF-beta-mediated fibrosis. Nat. Commun..

[140] Fathke C., Wilson L., Shah K., Kim B., Hocking A., Moon R., Isik F. (2006). Wnt signaling induces epithelial differentiation during cutaneous wound healing. BMC Cell Boil..

[141] Mori H., Yao Y., Learman B.S., Kurozumi K., Ishida J., Ramakrishnan S.K., Overmyer K.A., Xue X., Cawthorn W.P., Reid M.A. (2016). Induction of Wnt11 by hypoxia and hypoxia-inducible factor-1alpha regulates cell proliferation, migration and invasion. Sci. Rep..

[142] Scheid A., Wenger R.H., Christina H., Camenisch I., Ferenc A., Stauffer U.G., Gassmann M., Meuli M. (2000). Hypoxia-regulated gene expression in fetal wound regeneration and adult wound repair. Pediatr. Surg. Int..

[143] Hsu Y.C., Pasolli H.A., Fuchs E. (2011). Dynamics between stem cells, niche, and progeny in the hair follicle. Cell.

[144] Blanpain C., Fuchs E. (2006). Epidermal stem cells of the skin. Annu. Rev. Cell Dev. Biol..

[145] Blanpain C., Lowry W.E., Geoghegan A., Polak L., Fuchs E. (2004). Self-renewal, multipotency, and the existence of two cell populations within an epithelial stem cell niche. Cell.

[146] Watt F.M., Kubler M.D., Hotchin N.A., Nicholson L.J., Adams J.C. (1993). Regulation of keratinocyte terminal differentiation by integrin-extracellular matrix interactions. J. Cell Sci..

[147] Rennert R.C., Sorkin M., Garg R.K., Gurtner G.C. (2012). Stem cell recruitment after injury: Lessons for regenerative medicine. Regen. Med..

